# Exploring the Radiation Damage of Vacancy‐Ordered Double Perovskites, ((NH_4_)_(1−*x*)_FA_*x*_)_2_SnBr_6_

**DOI:** 10.1002/cphc.202500370

**Published:** 2025-11-04

**Authors:** Prajna Bhatt, Theo Stucky de Quay, Yuhan Liu, Tim Evans, Orfhlaith McCullough, Nathalie K. Fernando, Curran Kalha, Robert G. Palgrave, Anna Regoutz

**Affiliations:** ^1^ Department of Chemistry University College London 20 Gordon Street London WC1H 0AJ UK; ^2^ The Electrochemical Innovation Lab Department of Chemical Engineering University College London London WC1E 7JE UK; ^3^ School of Human Sciences Science Centre London Metropolitan University London N7 7DD UK; ^4^ Inorganic Chemistry Laboratory Department of Chemistry University of Oxford South Parks Road Oxford OX1 3QR UK; ^5^ Present address: Istituto Officina dei Materiali (IOM)‐CNR, Laboratorio TASC, in Area Science Park S.S.14, Km 163.5 Trieste 34149 Italy

**Keywords:** correlation analysis, halide perovskites, radiation damage, X‐ray photoelectron spectroscopy

## Abstract

Degradation studies of the ASnX_3_ perovskites to the A_2_SnX_6_ vacancy‐ordered double perovskite form have been well researched, but little is known about how A_2_SnX_6_ compounds degrade. Herein, a new double perovskite, FA_2_SnBr_6_ (FA = CH(NH_2_)_2_), is synthesized and characterized by using X‐ray diffraction and X‐ray photoelectron spectroscopy. FA_2_SnBr_6_ is found to crystallize in the *P*2_1_/*n* monoclinic space group and shows the ability to form single‐phase, solid solutions with another double perovskite (NH_4_)_2_SnBr_6_. Solid solutions of ((NH_4_)_(1−*x*)_FA_*x*_)_2_SnBr_6_, where *x* = 0.03, 0.04, 0.06, and 0.09, are produced, and X‐ray radiation damage is investigated to qualitatively and quantitatively uncover degradation mechanisms. Correlation analysis, a new statistical analytical method for X‐ray photoelectron spectra, is used to fortify peak fitting models. Comparing shifts in binding energies and relative atomic quantities between two elements constructs phase models from different spectral environments to understand the degradation of these compounds. In each radiation damage experiment, great consideration must be taken to combine the chemistry of the system and the physical process of photoelectron emission with the quantitative phase models formed. Overall, the presence of ammonium in the double perovskite enhances the stability of the compound to X‐ray irradiation.

## Graphical abstract

Incident X‐ray photons *hν* on a newly reported vacancy‐ordered double perovskite FA_2_SnBr_6_ results in damage of the compound. This is seen over increasing X‐ray dose using X‐ray photoelectron spectroscopy.

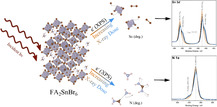

## 1 Introduction

Compounds with the formula A_2_BX_6_, where A is a monovalent cation, B is a tetravalent cation, and X is a halide, have been studied for decades and are undergoing a resurgence of interest due to their relation to the halide perovskite systems, ABX_3_. A_2_BX_6_, also referred to as the vacancy‐ordered double perovskite form, can be considered as an ABX_3_ perovskite with occupation of only half of the B‐sites in the structure. A_2_BX_6_ commonly adopts the cubic Fm$\bar{3}$m structure and can maintain this across a wide composition range.^[^ ^1^ ^]^ The high symmetry of the average structure conceals some intriguing features of structural chemistry, for example, anharmonicity in octahedral rotation, preferential ordering of halide ions, and cation rattling.^[^ ^2^, ^3^, ^4^ ^]^

A_2_BX_6_ compounds have become relevant in the field of perovskite solar cells, both as solar absorbers in their own right and as degradation products of halide perovskites. This includes tin halide perovskites ASnX_3_, which are promising lead‐free absorber materials.^[^ ^5^, ^6^ ^]^ Their extended charge carrier lifetimes, defect tolerance, and carrier diffusion length make these materials interesting for photovoltaic applications.^[^ ^7^, ^8^, ^9^ ^]^ However, ASnX_3_ suffers greatly from degradation due to the instability of the Sn (II) state, resulting in shorter carrier lifetimes. The instability has been studied by looking at the degradation of ASnX_3_, where A = methylammonium (CH_3_NH_3_, MA) or formamidinium (CH_3_(NH_2_)_2_, FA), in both ambient and inert conditions, by application of heat or pressure and analyzed using X‐ray diffraction (XRD) and solid‐state nuclear magnetic resonance (SS‐NMR) spectroscopy.^[^ ^10^, ^11^, ^12^ ^]^

An exemplar study by Kubicki et al. previously explored ex situ thermal degradation of ASnX_3_, where A = MA, FA, CS and X = Br, I, via Sn SS‐NMR. The work concluded, in particular, that FASnBr_3_ decomposes to FA_2_SnBr_6_, which is well established for other tin‐perovskites.^[^ ^13^, ^14^ ^]^ For the case of FA, the double perovskite has not been previously studied. No reported structure of FA_2_SnBr_6_ exists, but SS‐NMR suggests that the double perovskite does form due to thermal degradation. The degradation of formamidinium compounds is of interest to study as formamidinium bromide (FABr) has been reported to decompose at temperatures slightly higher than ambient conditions (323 K), to ammonium bromide NH_4_Br.^[^ ^15^ ^]^ Thus, while the degradation of FASnBr_3_ is well understood, studying the degradation of a possible FA_2_SnBr_6_ is not. Studies on it can help in understanding what can happen at the A‐site. Here, a detailed degradation study of the FA cation can add to the understanding of FASnBr_3_ as products from prolonged degradation. To study FA_2_SnBr_6_, it was essential to synthesize, confirm a crystal structure, and understand the chemical environments associated with the compound prior to any external degradation. This work reports the single crystal structure of FA_2_SnBr_6_, along with a comprehensive photoelectric spectroscopic study on FA_2_SnBr_6_, (NH_4_)_2_SnBr_6_, and solid solutions thereof, ((NH_4_)_(1−*x*)_FA_*x*_)_2_SnBr_6_.

X‐ray degradation by X‐ray photoelectron spectroscopy (XPS) of ((NH_4_)_(1−*x*)_FA_*x*_)_2_SnBr_6_ was undertaken to understand the degradation of FA_2_SnBr_6_, in particular, how the FA ion degrades in different compounds. Similar photoelectron spectroscopy‐based studies have studied degradation before.^[^ ^16^, ^17^, ^18^ ^]^ To understand the degradation mechanism both qualitatively and quantitatively, XPS correlation analysis has been applied to identify and understand phases that arise during irradiation.^[^ ^19^ ^]^ This newly established analytical method utilizes peak fitting procedures to understand multi‐level spectroscopic datasets.

## 2 Results and Discussion

### 2.1 Single‐Crystal Structure of FA_2_SnBr_6_

The structure of FA_2_SnBr_6_ was determined by single‐crystal X‐ray diffraction (SCXRD). Single crystals of FA_2_SnBr_6_ appear pale yellow in color, with no obvious morphology. From the analysis of SCXRD data collected at 280 K, the crystal structure of FA_2_SnBr_6_ was determined to be monoclinic with the *P*2_1_/*n* space group. The resolved lattice parameters are summarized in **Table** **1**. Unlike other reported A_2_SnBr_6_ compounds (A = Cs, Rb, NH_4_, K) and FA_2_SnI_6_,^[^ ^20^ ^]^ FA_2_SnBr_6_ deviates from the K_2_PtCl_6_ cubic structure (Fm$\bar{3}$m) at room temperature.^[^ ^3^, ^21^ ^]^ However, the *P*2_1_/*n* space group has been reported for several other A_2_BX_6_ compounds, such as K_2_TeX_6_ (X = Br, I).^[^ ^22^, ^23^ ^]^

**Table 1:** Unit cell parameters obtained from the Rietveld refinement of SCXRD data collected at 280 K of the monoclinic *P*2_1_/*n* FA_2_SnBr_6_. *a*, *b,* and *c* are the lattice parameters, *α*, *β,* and *γ* are the unit cell angles, *V* is the unit cell volume, and *Z* is the number of formula units in the unit cell.

FA_2_SnBr_6_			
*a*/Å	7.55098(1)	*α*/°	90
*b*/Å	12.71242(8)	*β*/°	103.14(0)
*c*/Å	8.31148(2)	*γ*/°	90
*V*/Å^3^	776.939(2)	*Z*	2

**Figure 1:**
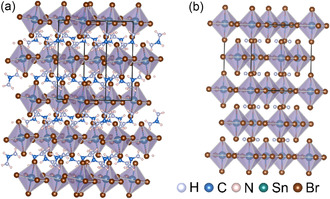
Crystal structures of selected tin vacancy‐ordered double perovskites, including a) the monoclinic FA_2_SnBr_6_ from this work, and b) the cubic (NH_4_)_2_SnBr_6_ from the Inorganic Crystal Structure Database (ICSD) with collection code 158954.^[^ ^21^ ^]^ FA_2_SnBr_6_ is aligned along the [010] and (NH_4_)_2_SnBr_6_ along the [001] Miller planes, made using the VESTA version 3 software suite.^[^ ^58^ ^]^ Brown atoms represent Br, pink atoms represent H, blue atoms represent C, purple atoms represent N, and teal atoms represent Sn. The light gray octahedra represent [SnBr_6_]^2−^ octahedra.

FA_2_SnBr_6_, in **Figure** **1**a, is composed of almost‐regular [SnBr_6_]^2−^ octahedra with bond angle variance of 0.192 deg^2^ and three near indistinguishable Sn–Br bond distances which are 2.6010, 2.6080, and 2.6027 Å. These are arranged in a form that can be considered a lower symmetry derivative of the cubic K_2_PtCl_6_ structure. The monoclinic axes are such that the *b*‐axis is along the [100] direction of the cubic aristotype, and the *a*‐ and *c*‐axes are along the [011] and [$0\bar{1}1$] directions, respectively. In the cubic structure, represented by (NH_4_)_2_SnBr_6_ in Figure 1b, the *C*_4_ axes of the octahedra lie along the cubic unit cell axes. In the monoclinic structure, rotations of the octahedra occur, which can be measured as the angle of the *C*_4_ axis of the octahedron away from the cubic axis of the aristotype. These angles are 10.3°, 10.9°, and 7.5° for the *a*‐, *b*‐, and *c*‐cubic axes, respectively, the tilts alternating in direction along the monoclinic *b*‐axis. These tilts are slightly greater than those reported for K_2_TeBr_6_ (7.1°, 6.9°, 8.7°).^[^ ^23^ ^]^ In addition, the monoclinic angle shows a greater deviation from 90° in FA_2_SnBr_6_ compared with K_2_TeBr_6_.

Considering the coordination of the bromide anions to the A‐site, the FA^+^ ion sits in an irregular 12‐coordinate cavity, with an average C‐Br distance of 4.173 Å, slightly larger than the Cs‐Br distances observed in cubic Cs_2_SnBr_6_ (4.119 Å). While the volume of the ABr_12_ cuboctahedron is almost identical in Cs_2_SnBr_6_ and FA_2_SnBr_6_, the significant distortion of the latter structure and the nonspherical nature of the FA^+^ ion allow a minimum C–Br distance of 3.658 Å. This is approximately the same as the N–Br distance in cubic (NH_4_)_2_SnBr6 (3.737 Å). Brown^[^ ^23^ ^]^ used the ABr_12_ cuboctahedron to explain the distortion of K_2_TeBr_6_ by considering the size of the A‐site ion relative to the cuboctahedral cavity, measured via a radius ratio, which for K_2_TeBr_6_ was 0.89. It was considered that small A‐site ions (represented by a radius ratio far below 1) would lead to distortion away from the cubic structure as octahedra rotated to optimize A–X bonding. In this case, the FA^+^ ion (if considered spherical with an effective radius of 2.56 Å following Kieslich et al.)^[^ ^24^ ^]^ is considerably larger than the cavity with a radius ratio using Brown's method of 1.47, causing a similar distortion. In reality, the FA^+^ ion is not spherical, and the extent of distortion of the cavity is likely to be affected by this.

Employing geometric modeling to understand the differences in the crystal structures of FA_2_SnX_6_ (X = Br, I),^[^ ^1^, ^24^ ^]^ the larger [SnI_6_]^2−^ octahedra allow the A‐site to sit in its crystallographic site compared to the analogous bromide compounds. The iodide ions in neighboring octahedra can contrariwise touch each other, unlike in the bromide where FA^+^ is too large, preventing this. Concurrently, if an X‐site cubic sublattice is modeled so that the halides touch as hard spheres, the FA^+^ ion fits in the sublattice for X = I but overlaps for X = Br, aligning with the experimental noncubic crystal system deduction. The aforementioned models previously applied to A_2_SnBr_6_ (A = Cs, Rb, NH_4_, K),^[^ ^1^ ^]^ described the same conclusion as FA_2_SnI_6_: the A‐site cations can position themselves within a cubic ordered X‐site sublattice, without any distortion.

The structure, as understood from SCXRD, was compared to data collected via powder X‐ray diffraction (PXRD). Using the unit cell parameters obtained from SCXRD analysis in Table 1, the Rietveld refinement of PXRD data is depicted in Figure S1, Supporting Information. A goodness of fit of 1.16 and *R*_w_ = 9.676% was achieved, demonstrating the same monoclinic structure for FA_2_SnBr_6_ in powdered form. The usage of SCXRD‐derived lattice parameters, matching well in a refinement of the FA_2_SnBr_6_ model to the PXRD obtained pattern, fortifies the conclusive monoclinic structure for the double perovskite. Since the aforementioned (NH_4_)_2_SnBr_6_ analogue is within the cubic K_2_PtCl_6_ crystal family, it remains to see what crystal system solid solutions of the FA‐ and NH_4_‐based double perovskites lie within, explored in the following section. These will be used to establish double perovskite systems containing both A‐sites as references for the following radiation damage studies.

### 2.2 Solid Solution Series of ((NH_4_)_(1−*x*)_FA_*x*_)_2_SnBr_6_

**Figure 2:**
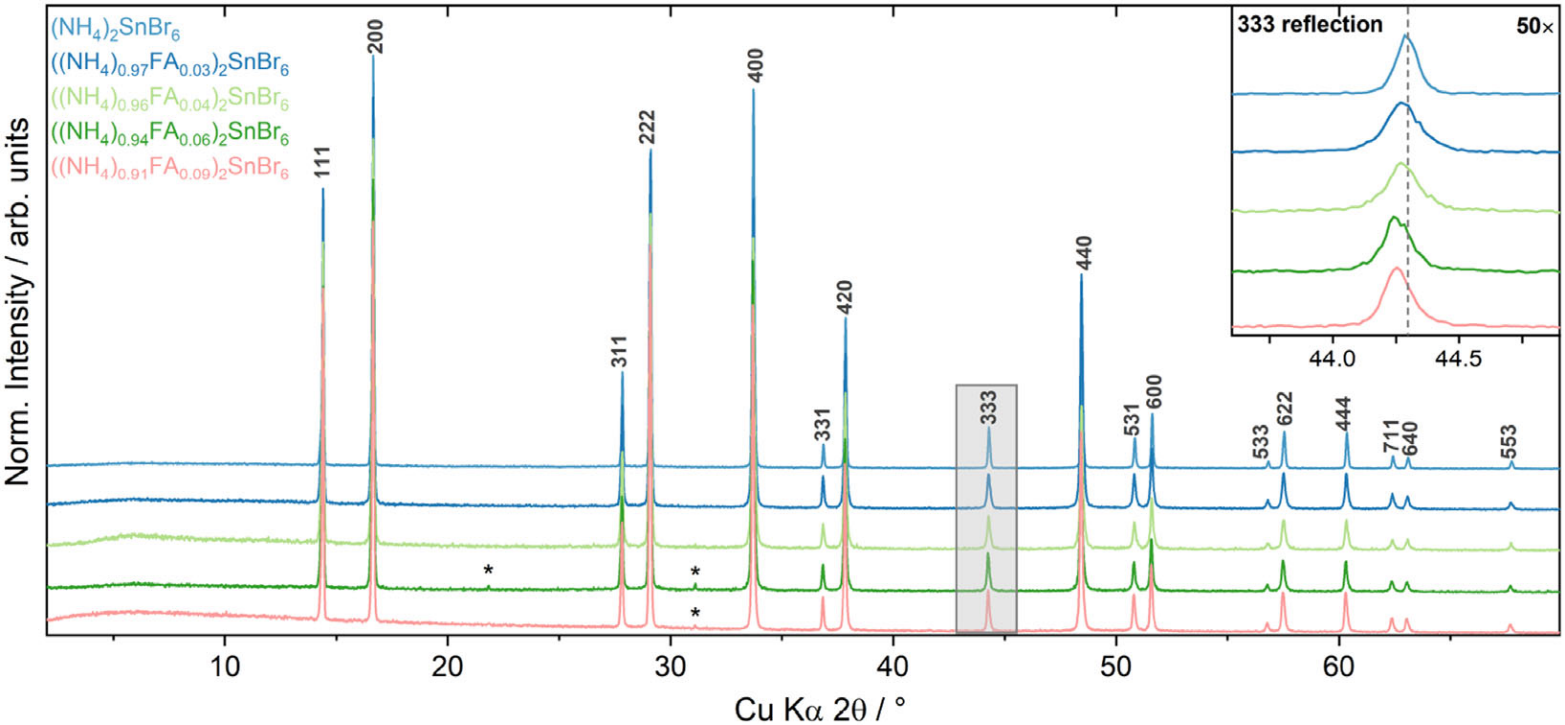
PXRD patterns of ((NH_4_)_(1−*x*)_FA_*x*_)_2_SnBr_6_ with *x* = 0, 0.03, 0.04, 0.06, and 0.09. The inset shows a 50× magnified view of the 333 reflection, highlighted with the light gray box between 42 and 47°. The gray dashed line in the inset marks the peak position of the undoped structure. Asterisks mark reflections from NH_4_Br.

To explore the crystalline structures of the solid solution series ((NH_4_)_(1−*x*)_FA_*x*_)_2_SnBr_6_, PXRD was conducted and is shown in **Figure** **2**. For 0 ≤ *x* < 0.1, patterns are isostructural to the Fm$\bar{3}$m (NH_4_)_2_SnBr_6_ double perovskite. No additional peaks were observed from secondary crystalline perovskite‐like phases, highlighting the formation of phase‐pure, doped double perovskites. Some patterns include reflections from NH_4_Br, a reactant used during synthesis. The inclusion of FA into the (NH_4_)_2_SnBr_6_ structure is proven by the shift in 2*θ* position of the diffraction patterns. The inset depicts this for the 333 reflection, wherein the gray dashed line marks the peak position of the undoped structure. A global shift of the 333 reflection to lower 2*θ* angles is observed upon addition of FA, indicating an increasing *d* spacing and unit cell size. Lattice expansion is expected as FA^+^ with an ionic radius of 2.53 Å is larger than ${\text{NH}}_{4}^{+}$ (ionic radius = 1.46 Å).^[^ ^24^ ^]^ Substitution of the larger formamidinium cation into (NH_4_)_2_SnBr_6_ is commensurate with the whole diffraction pattern shifting to lower angles relative to the undoped (NH_4_)_2_SnBr_6_. This confirms that insertion of FA^+^ into the Fm$\bar{3}$m space group structure has been successful.

**Figure 3:**
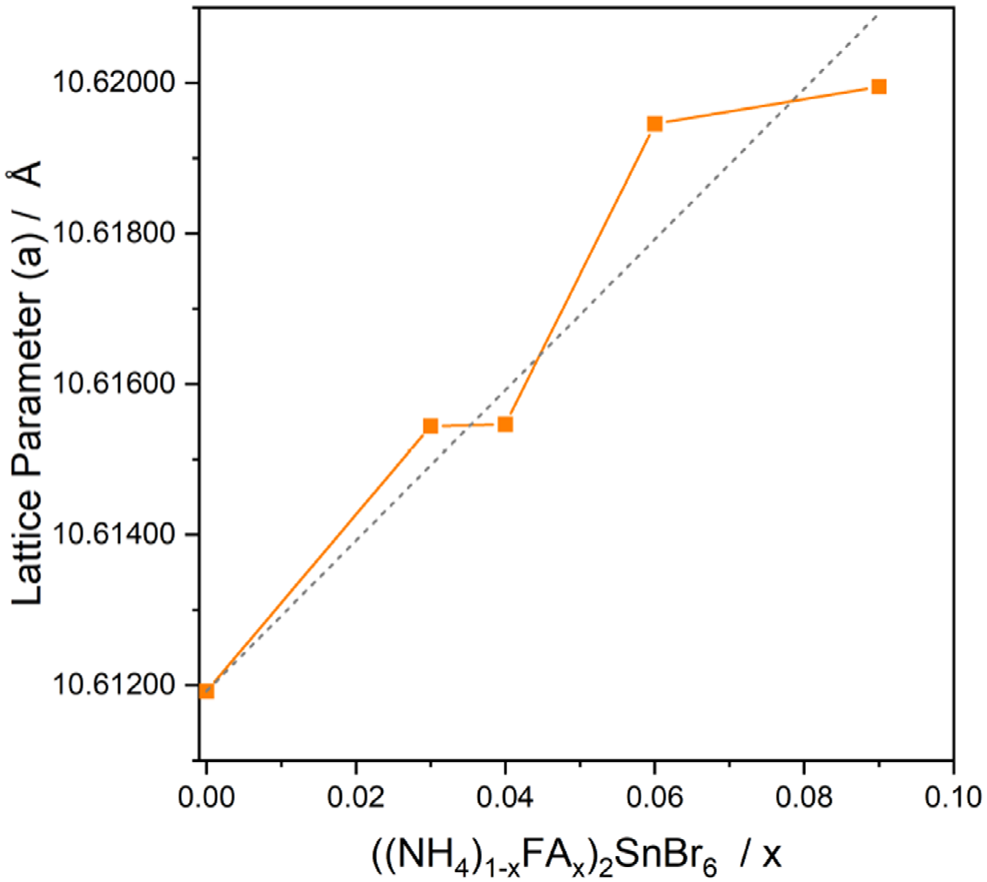
Variation of lattice parameter *a* in the cubic ((NH_4_)_(1−*x*)_FA_*x*_)_2_SnBr_6_ series for *x* between 0 and 0.1. Values were derived from Pawley refinement using the TOPAS software package.^[^ ^48^ ^]^ The gray dotted line with a gradient of one represents Vegard's law.^[^ ^26^ ^]^

The lattice parameter *a* from the PXRD data obtained by Pawley refinement^[^ ^25^ ^]^ from the TOPAS‐Academic software is illustrated in **Figure** **3**. The plot shows an increase in *a* when the FA content increases. The relationship approximately follows Vegard's law (represented by the gray dashed line in the Figure), with the largest increase being ≈0.1% for *a* relative to *x* = 0.^[^ ^26^ ^]^ Despite the successful doping of the double perovskite on the A‐site, there is no observable trend in the optical bandgap of these materials as seen in Figure S4, Supporting Information. This is likely due to the fundamental interband transition involving orbitals from the [SnBr_6_]^2−^ octahedra, and therefore, changing A‐site ions do not greatly influence the bandgap.^[^ ^27^ ^]^ When the FA content, *x* > 0.1, mixed phase patterns were observed and the monoclinic FA_2_SnBr_6_ phase was detected in addition to the cubic phase. Formation of ${\text{NH}}_{4}^{+}$‐doped FA_2_SnBr_6_ was also attempted, and failed to form single‐phase double perovskites, see Figure S2, Supporting Information.

**Figure 4:**
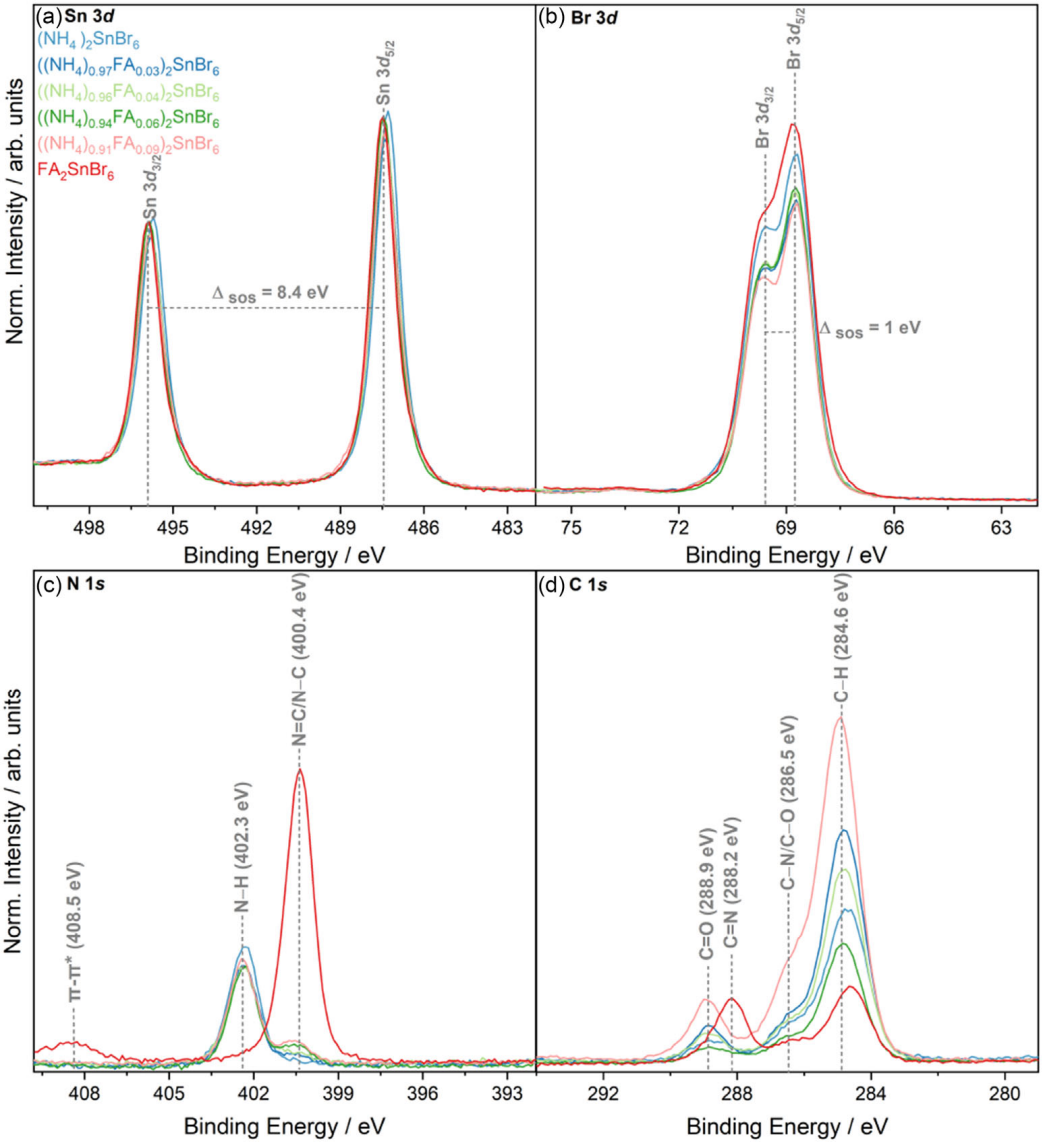
Core‐level X‐ray photoelectron spectra of ((NH_4_)_(1−*x*)_FA_*x*_)_2_SnBr_6_, where *x* = 0, 0.03, 0.04, 0.06, 0.09, including a) Sn 3*d*, b) Br 3*d*, c) N 1*s*, and d) C 1*s*. Data are normalized to the peak area of the respective Sn 3*d*_5/2_ peak.

XPS was conducted on the powdered samples to understand how chemical environments and elemental compositions vary across the solid solution series. Principal core levels are shown in **Figure** **4**, and the surveys are shown in Figure S5,Supporting Information. In the case of FA_2_SnBr_6_, the difference in binding energy positions (ΔBE) between the Sn 3*d*_5/2_ against Br 3*d*_5/2_ and N 1*s* core levels against Br 3*d*_5/2_ are commensurate with ΔBE from XPS reported for MA_2_SnBr_6_^[^ ^28^ ^]^ 418.7 eV for Sn 3*d*_5/2_ and 331.6 eV for N 1*s*, respectively. For the ammonium‐containing compounds, the ΔBE between the high intensity N 1*s* and Br 3*d*_5/2_ (333.5 eV) is similar to (NH_4_)_2_TcBr_6_.^[^ ^29^ ^]^ The similarity in ΔBE of core levels of those reported here to literature confirms the +1 oxidation states of the A‐site cations, the Sn (IV) B‐site, and the Br^−^ ion. The ΔBE value approach is used here as absolute values for semiconductor materials like the double perovskites can often cause misidentification of the chemical states.^[^ ^28^, ^30^ ^]^ The core levels of the doped and pure ammonium double perovskites were modeled with pseudo‐Voigt peaks employing a 70% Gaussian and 30% Lorentzian contribution over a Shirley background. An extended discussion of the peak fitting approach is included in the Supporting Information, Section S7. From the peak fit analysis of FA_2_SnBr_6_ it is clear that the Sn 3*d* shown in Figure 4a shows some asymmetry toward its lower binding energy (BE) side, hinting at radiation damage occurring during the experiment leading to partial reduction of the sample, which will be explored in detail in the following Sections. The Br 3*d* core level appears similar across the solid solution series (see Figure 4b). Across the series, the full width at half maximum (FWHM) of each singlet peak in the Br 3*d* doublet is 1.04 eV, indicating a dominant singular environment of the halide under the experimental conditions for this class of compounds, seen from a combination of lifetime and instrumental broadening contributing to the line shape.^[^ ^3^, ^28^ ^]^

The main differences are observed in the N 1*s* and C 1*s* core‐level spectra, see Figure 4c,d, respectively, which show environments from the A‐site cations. The formamidinium ion (C=N/C–N) at ≈400.5 eV and the ammonium ion (N–H) at ≈402.3 eV are well‐resolved in the N 1*s* spectra. Both single‐phase samples have one symmetric N 1*s* peak, while two environments are observed for the doped samples. Scaling the N 1*s* peak to the Sn 3*d*_5/2_ area shows the presence of four N atoms per one Sn atom in FA_2_SnBr_6_, compared with only two N atoms in (NH_4_)_2_SnBr_6_. The lower BE position for FA‐doped samples matches with pure FA_2_SnBr_6_.

The increasing intensity of the environment attributed to FA shows that doping has occurred across the series. FA_2_SnBr_6_ also has a π–π* interaction at ≈408.5 eV, from the nature of the bonding in the FA cation, arising from the N=C bonding.^[^ ^31^ ^]^ Peak fitting of the two nitrogen environments was done to quantify the formamidinium concentration achieved. The FA concentration (*x*) shows reasonable agreement with flash combustion elemental analysis shown in Figure S3, Supporting Information. Generally, XPS tends to overestimate the amount of FA relative to elemental analysis with the most significant discrepancy at *x* = 0.04 of ≈7 rel. at%. These are attributed to the surface sensitivity of XPS, where arbitrary spot selection may affect the estimation of *x*. As *x* values between the surface (XPS) and bulk (combustion analysis) elemental quantifications are minute, this work uses the bulk elemental quantifications for *x* in all further discussions.

In Figure 4d, the C 1*s* spectrum for FA_2_SnBr_6_ displays the C–N (286.5 eV) and C=N (288.2 eV) environments as expected, agreeing with observations of FA in FAPbI_3_.^[^ ^32^ ^]^ The C=O and C–O peaks in all samples are attributed to the ambient environment exposure. The C–H contribution arises from both adventitious carbon from ambient exposure and the FA cation. Additionally, the C–N environment overlaps with the assigned C–O adventitious peak. As such, overlapping or indistinguishable C 1*s* environments mean that quantification using C 1*s* is inappropriate for FA‐based compounds. O 1*s* has asymmetric peaks with multiple chemical environments in Figure S6, Supporting Information. Additionally, C–O is present in (NH_4_)_2_SnBr_6_, where no C–N environment is expected. Thus, this environment is labeled here as C–O/C–N to avoid over‐interpretation of the C 1*s* spectra where features are not well resolved.

So far, diffraction, elemental, and spectroscopic studies show that a solid solution series between the monoclinic FA_2_SnBr_6_ and cubic (NH_4_)_2_SnBr_6_ is possible. The doped variant forms within the $\text{Fm}\bar{3}m$ cubic structure, and the maximum *x* value is 0.09. Using selected compounds from the solid solution series, the next sections aim to explore their radiation sensitivity under characterization conditions. Evolving changes during XPS measurements are studied with correlation analysis to identify existing and new chemical phases due to photoemissive degradation.

### 2.3 Radiation‐Induced Damage of ((NH_4_)_(1−*x*)_FA_*x*_)_2_SnBr_6_

#### 2.3.1 FA_2_SnBr_6_, *x* = 1

**Figure 5:**
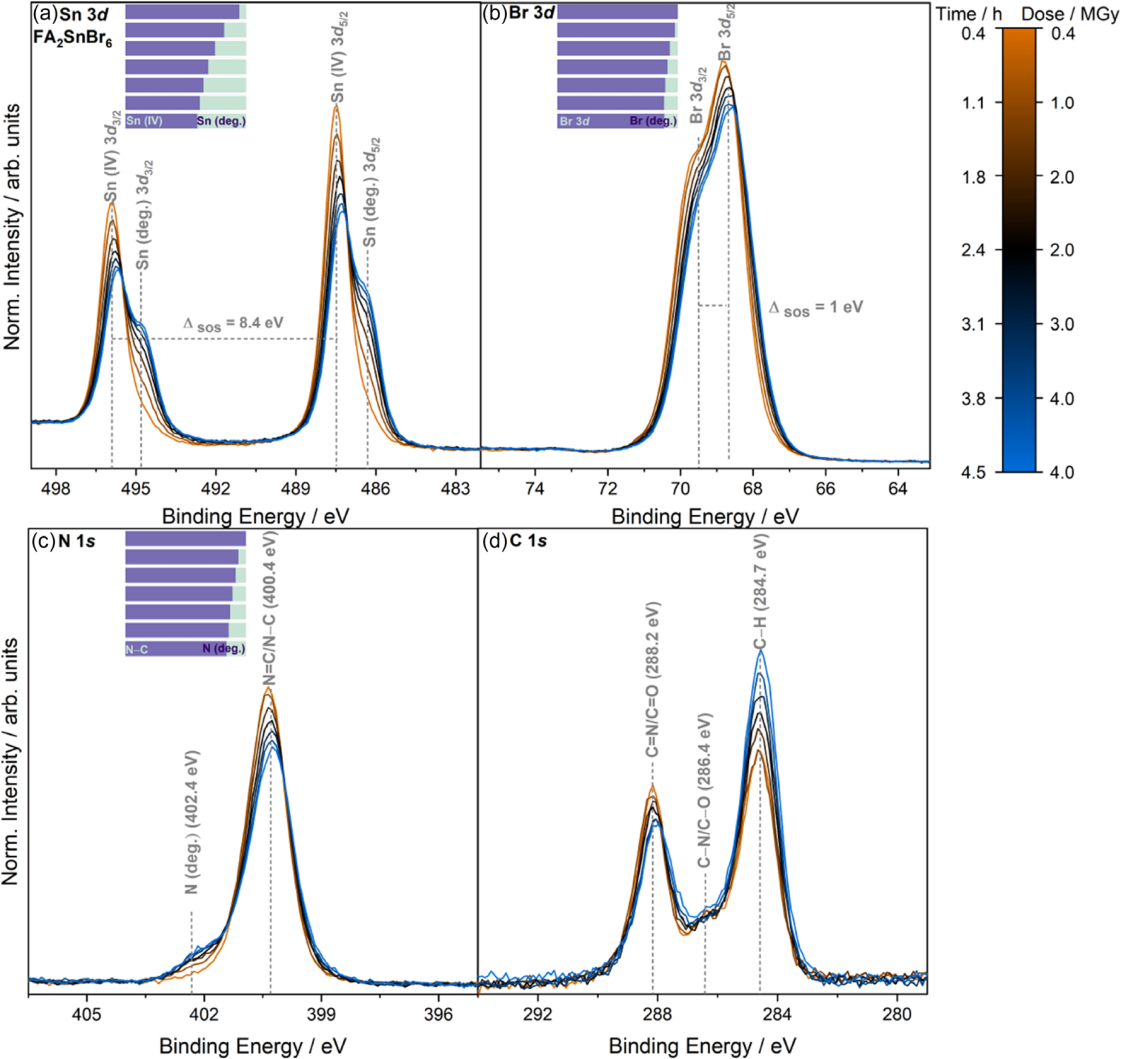
Core‐level X‐ray photoelectron spectra of FA_2_SnBr_6_ as a function of X‐ray dose, including a) Sn 3*d*, b) Br 3*d*, c) N 1*s*, and d) C 1*s*. The color bar legend at the right shows the cumulative measurement time and the calculated X‐ray dose from the AD‐WC using RADDOSE‐3D. The time given represents the end of each measurement period, with each data acquisition taking ≈0.42 h. The gray dotted lines and BE values shown correspond to the positions of the visually discernible spectral features. The (deg.) label is used to describe spectral features that appear in core‐level spectra as a result of irradiation. Data are normalized to the peak area of Sn 3*d*_5/2_ from the spectrum after exposure to 0.4 MGy of X‐ray dose after subtraction of a linear background. An indication of selected chemical species ratios as determined from peak fit analysis is included as an inset bar chart on the top left of each subfigure.

Distinct changes are observed in the core‐level spectra of FA_2_SnBr_6_ during XPS characterization as a function of X‐ray dose (rounded to the nearest whole number, in MGy), as shown in **Figure** **5**. The similarity between the dose absorbed and the duration of the experiment is purely coincidental and is explained further in Section S4, Supporting Information of the RADDOSE‐3D calculations. The rounding of dose values to the nearest whole number is not indicative of a stagnant dose over time; this has been employed in accordance to other such damage studies via XPS.^[^ ^16^, ^33^ ^]^ The most noticeable changes in the peak shapes of the core levels include the formation of a lower BE shoulder (≈1 eV below the peak maximum, labeled as Sn (deg.)) in the Sn 3*d* doublet (Figure 5a). Here (deg.) is used to categorize a spectral feature corresponding to a chemical environment that arose from RADDAM. Sn (deg.) is representative of the photoreduction of Sn (IV), known for tin‐based double perovskites.^[^ ^34^ ^]^ The Br 3*d* peak (Figure 5b) shows comparatively little change, with only a slight broadening of the doublet occurring upon radiation. N 1*s* (Figure 5c) shows an increasing intensity of the higher BE feature at ≈402.4 eV, labeled N (deg.). The BE position of the N (deg.) feature is associated with the (N–H) environment in ammonium‐based perovskites. This feature was also noted in other work in the literature that explored the X‐ray irradiation of FAPbX_3_, X = Br, I.^[^ ^35^, ^36^, ^37^, ^38^ ^]^ For C 1*s*, as shown in Figure 5d, there is an overall decrease in the intensity of the C=N/C=O environment while the C‐H environment intensity increases. Additionally, the rates of change of these two environments are unequal—with C–H increasing faster than C=N/C=O decreasing during irradiation.

To understand the chemical phases that exist before and after X‐ray exposure in FA_2_SnBr_6_, the concept of correlation analysis for photoelectron spectroscopy is applied here.^[^ ^19^ ^]^ Briefly, this is an analytical procedure that uses parameters typically obtained from peak fitting procedures, including BE position and atomic quantification, to construct phase models within a chemically stable system measured by XPS. In this work, correlation analysis is applied to understand the formation of new chemical phases over the course of a radiation damage XPS experiment. To conduct correlation analysis, peak fitting was applied to all spectra shown in Figure 5 and is detailed in Section S7, Supporting Information. Chemical environments that inherently belong to FA_2_SnBr_6_, as described in the previous Section, and any new environments that appear upon X‐ray exposure were fitted. These peak fits must be physically meaningful, including the selection of appropriate line widths and shapes, spin‐orbit splitting (SOS), and photoionization cross sections. Correlation analysis uses extracted BE peak positions (in eV) and elemental compositions, also called ‘phase fractions’ (in rel. at%), to study pairwise correlations between different chemical environments, hereafter referred to as “phase models”. The details of how correlation analysis can be employed are explained elsewhere.^[^ ^19^ ^]^ To summarize, the BE and phase fraction correlations are expected to be linear if the environments belong to the same chemical phase. For all compounds in this work, correlation analysis avoids using the C 1*s* core level due to the difficulties in separating chemical states that arise from the sample and adventitious species discussed in the section above for the doped series. As nitrogen is present in all double perovskites, N 1*s* is sufficient for constructing chemical phases.

**Figure 6:**
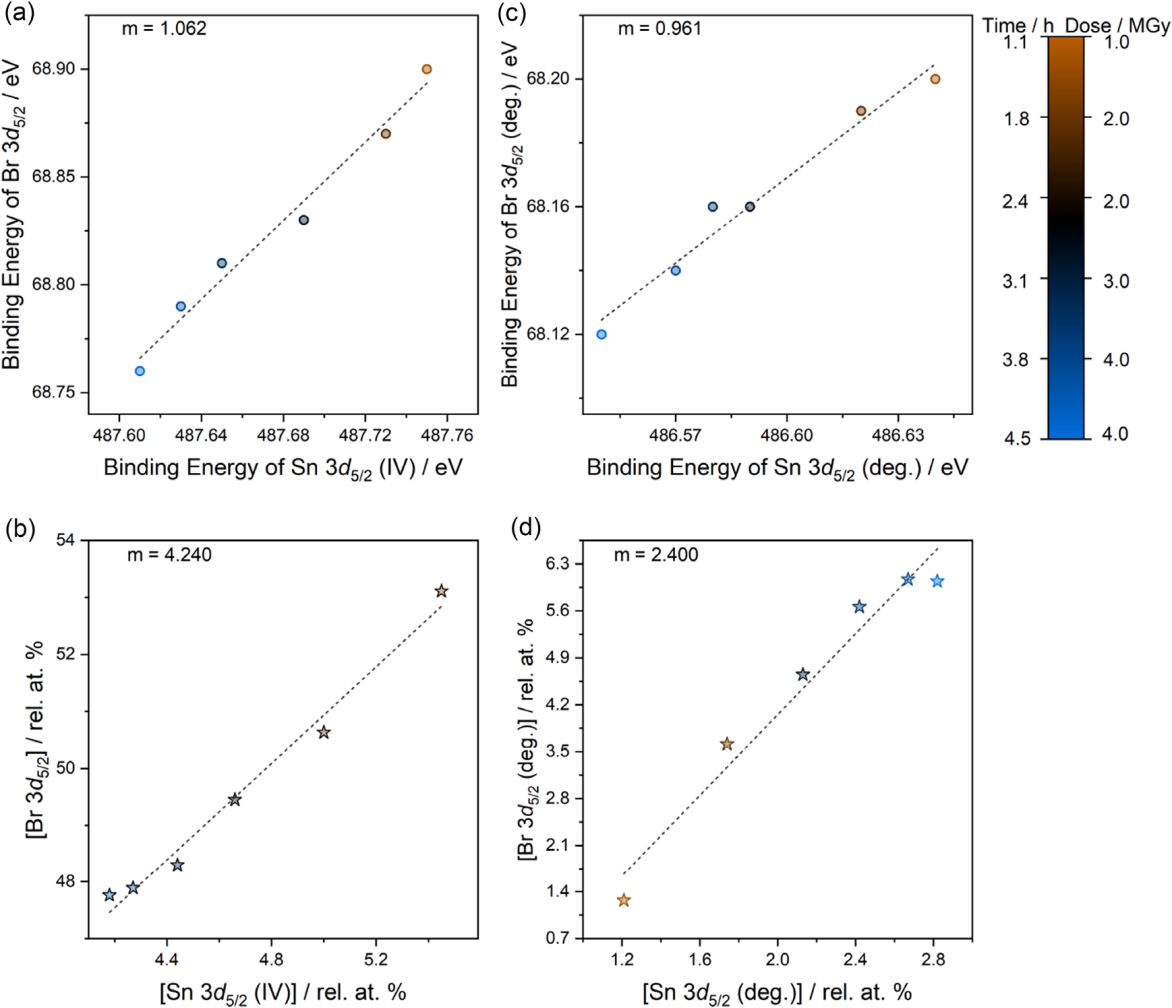
Correlation graphs a,c) BE positions and b,d) phase fractions from the XPS core‐level dataset of FA_2_SnBr_6_ radiation damage experiment. Correlations are depicted for degraded peaks in XPS that have assigned as a,b) Sn 3*d*_5/2_ (IV) against Br 3*d*_5/2_, and c,d) Sn 3*d*_5/2_ (deg.) against degraded Br 3*d*_5/2_ (deg.). Correlations of their BE positions and phase fractions are shown as a function of X‐ray exposure, and values of the gradient (*m*) for these correlations are provided in each subfigure.

Differences in the BE positions can be used to determine which elements belong to a phase with a fixed chemical composition. This assumes that chemical environments within the same phase will have a constant BE separation. While their absolute BE may change due to charging or Fermi level alteration (e.g., due to defect formation),^[^ ^39^ ^]^ the BE difference between elements in the same compound is close to constant under most circumstances. Therefore, chemical environments in the same phase should exhibit unitary relationships between BE changes, i.e., the gradient of the correlation graph of BE positions should be equal to one. For FA_2_SnBr_6_, BE correlations were first calculated for peak environments previously ascribed to the double perovskite. This allows for the confirmation of a phase model that is known and expected for this experiment, which has been proven by additional characterization methods such as XRD. Additionally, by confirming the FA_2_SnBr_6_ phase using correlation analysis, other systematic pairwise relations can be extracted for features that appear due to radiation‐induced damage. Thus, Sn 3*d* (IV), Br 3*d*, and the FA‐related N 1*s* core‐level environments should show unitary correlations if these environments have been assigned and modeled correctly. **Figure** **6**a shows the BE correlation between the Br 3*d*_5/2_ and Sn 3*d*_5/2_ (IV) features. The almost unitary (*m* = 1.062) relation between BEs of Sn (IV) 3*d*_5/2_ and Br 3*d*_5/2_ and for the (N=C/N–C) N 1*s* peak at 400.4 eV against the Sn and Br peaks (see Table S4,Supporting Information for all BE correlations) show peak fitting appropriately assigned peaks that belong to FA_2_SnBr_6_. There appears to be no obvious differential charging or unoptimized peak fitting in this dataset, as correlations for the double perovskite environments are linear.

Correlations of phase fractions can then be used to construct a sensible chemical phase for a given system. This is shown in Figure 6b and Table S4, Supporting Information summarizes all phase fractions for FA_2_SnBr_6_. This analysis does not consider the first collected spectrum (after exposure to 0.4 MGy), where the adventitious C and O environments dominate, affecting quantification. The intensity of adventitious species reduces over time, as shown in Figure S12, Supporting Information, showing the change in the elemental compositions from peak fit analysis of the core‐level spectra. The carbonaceous surface‐sensitive species desorb over time due to continual X‐ray and flood gun exposure. Simultaneously, the removal of the irradiated adventitious species to the ultra‐high vacuum (UHV) environment in the analysis chamber is known for XPS experiments.^[^ ^40^ ^]^ Hence, phase fractions calculated after the first spectrum better represent the pristine double perovskite system. For FA_2_SnBr_6_, the nominal Br:Sn (IV) phase fraction should be six, and the calculated phase fraction here is *m* = 4.240. This difference between the nominal and calculated phase fraction can be explained by the reduction in the rel. at% of Br 3*d* relative to Sn and N core levels, shown in Figure S12a, Supporting Information. The amount of Br decreases continually due to the loss of Br in the form of Br_2_ gas, an effect well known to occur during XPS for halide perovskite compounds.^[^ ^41^, ^42^, ^43^ ^]^ Considering this, the differences in the nominal and calculated phase fractions are unsurprising, and combined with the BE correlation, they indicate that the Br and Sn (IV) environments both belong to the double perovskite, as expected.

For the A‐ and X‐site elements, in Table S4 (Supporting Information), the phase fraction of N:Sn (IV) is 2.804, which nominally should be 4, as the FA^+^ ion contains two nitrogen atoms. Like Br, the N content decreases over the course of the experiment, again explaining the difference between phase fractions. These correlations, in parallel with BE, prove that the phase for FA_2_SnBr_6_ has been well constructed from peak fitting. It also shows that X‐ray irradiation leads to a degradation of the double perovskite that can, at times, cause the formation of gaseous compounds. This is also noted for thermal degradation of iodide and bromide tin perovskites elsewhere.^[^ ^10^, ^11^ ^]^ Here, correlation analysis produces conclusions with chemically intuitive approaches to the peak fitting of spectroscopic data. Using correlation analysis, the highest intensity spectral features have been identified as the double perovskite, which has also been independently proven by other analytical methods, such as SCXRD and PXRD in the previous Sections of this work. Thus, correlations can now be used to identify chemical phases that result from irradiation, namely the Sn (deg.), Br (deg.), and N (deg.) environments shown in the peak fits of XPS spectra of FA_2_SnBr_6_ in Figure S7, Supporting Information.

For two of these irradiation‐related environments, Figure 6c shows that the BE correlation between Sn (deg.) and Br 3*d*_5/2_ (deg.) is positive and linear (*m* = 0.961) with a phase fraction of 2.400 between Br (deg.):Sn (deg.). This indicates that Sn (deg.) and Br (deg.) belong to a singular chemical phase, and a phase fraction of ≈2 can indicate that SnBr_2_ forms. Photoreduction of the B‐site in perovskite‐related structures is well known,^[^ ^17^, ^18^ ^]^ and a chemically stable form of a reduced Sn (IV) species would be Sn (II). Hence, SnBr_2_ is the most chemically plausible species arising from degradation. For N 1*s* (deg.), BE correlations between N 1*s* (deg.) and the other degraded environments are negative: −1.17 with Sn (deg.) and −0.984 Br (deg.). This means the FA^+^ ion degrades independently of the Sn and Br of FA_2_SnBr_6_. The degradation of formamidinium ions to ammonia, formamidine, and *sym*‐triazine is well known from thermal degradation.^[^ ^37^, ^38^ ^]^ Of the three, as a result of an increasing temperature, the formation of ammonia gas occurs first, followed by formamidine, and lastly, *sym*‐triazine. The absence of positive linear correlations between N (deg.) and other elements present in FA_2_SnBr_6_ suggests that the degradation of FA follows this mechanism. The formation of NH_3_(g) is supported by the differences between the nominal and calculated phase fractions of N:Sn (IV) for the double perovskite, discussed previously. The measured N (deg.) feature is likely to belong to formamidine. The relative position of the N (deg.) peak aligns with the N–H species seen from XPS in (NH_4_)_2_SnBr_6_ in the previous section. Formamidine is the only compound with a N–H environment that will remain on a sample surface and be measured by XPS. *Sym*‐triazine only has C=N environments, while formed gaseous ammonia species is removed from the sample surface under UHV conditions.

Correlation analysis has independently identified the primary FA_2_SnBr_6_ phase and has shown that the degradation of the double perovskite results in the formation of SnBr_2_, formamidine, NH_3_(g), and Br_2_(g) from exposure to X‐ray radiation. A similar degradation pathway for the B‐ and X‐sites was observed for lead‐based perovskites under X‐ray radiation.^[^ ^44^ ^]^ The peak fitting models applied were able to produce phase models that align with evidence from previous experiments on double perovskite compounds that employed experimental degradation methods other than XPS. Clearly, FA degradation is independent of the other elements in the double perovskite. To extend this understanding to other compounds in the solid solution series, where both FA and NH_4_ ions are present, it is necessary to understand the degradation of (NH_4_)_2_SnBr_6_.

#### 2.3.2 (NH_4_)_2_SnBr_6_, *x* = 0

**Figure 7:**
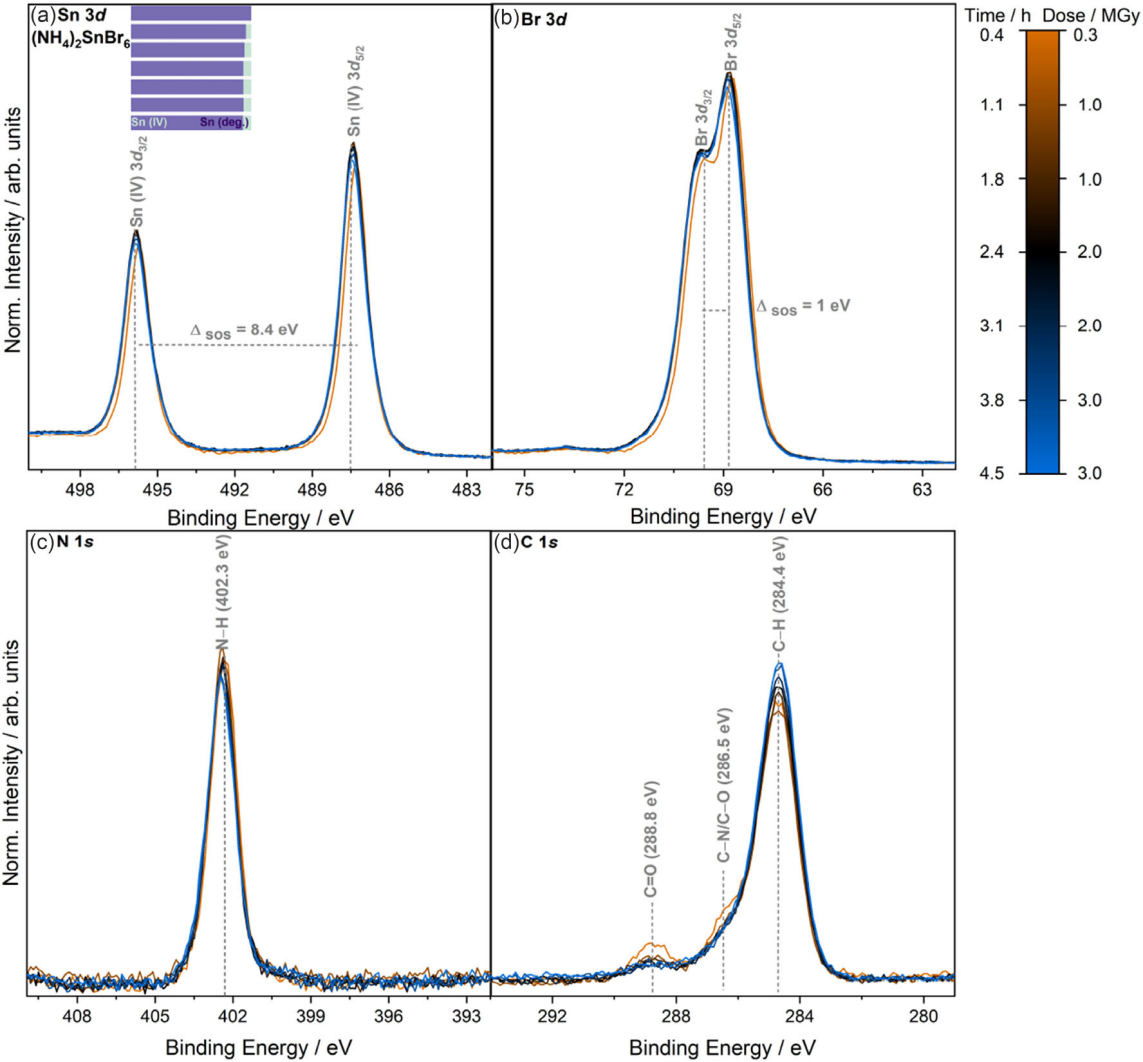
Core‐level X‐ray photoelectron spectra of (NH_4_)_2_SnBr_6_ as a function of X‐ray dose, including a) Sn 3*d*, b) Br 3*d*, c) N 1*s*, and d) C 1*s*. The color bar legend at the right shows the cumulative measurement time and the calculated X‐ray dose from the AD‐WC using RADDOSE‐3D. The time given represents the end of each measurement period, with each data acquisition taking ≈0.42 h. The gray dotted lines and BE values shown correspond to the positions of the visually discernible spectral features. The (deg.) label is used to describe spectral features that appear in core‐level spectra as a result of irradiation. Data are normalized to the peak area of Sn 3*d*_5/2_ from the spectrum after exposure to 0.3 MGy of X‐ray dose after subtraction of a linear background. An indication of selected chemical species ratios as determined from peak fit analysis is included as an inset bar chart on the top left of each subfigure.

Contrasting to FA_2_SnBr_6_, the (NH_4_)_2_SnBr_6_ core‐level spectra do not show any obvious changes during X‐ray irradiation as shown in **Figure** **7**. No additional chemical state peaks are observed outside of the FWHM of the main chemical state core lines. The difference in X‐ray dose incident on the compound (0.3 MGy) is not significant enough to explain the absence of radiation‐induced changes. The X‐ray dose difference arises from the reduction in the number of atoms between the ammonium (16) and formamidinium analogues (27), see more details in Section S4, Supporting Information about the RADDOSE‐3D parameters. This results in a higher total X‐ray absorption for FA_2_SnBr_6_ compared to (NH_4_)_2_SnBr_6_, and thus higher X‐ray dose values.^[^ ^45^ ^]^

**Figure 8:**
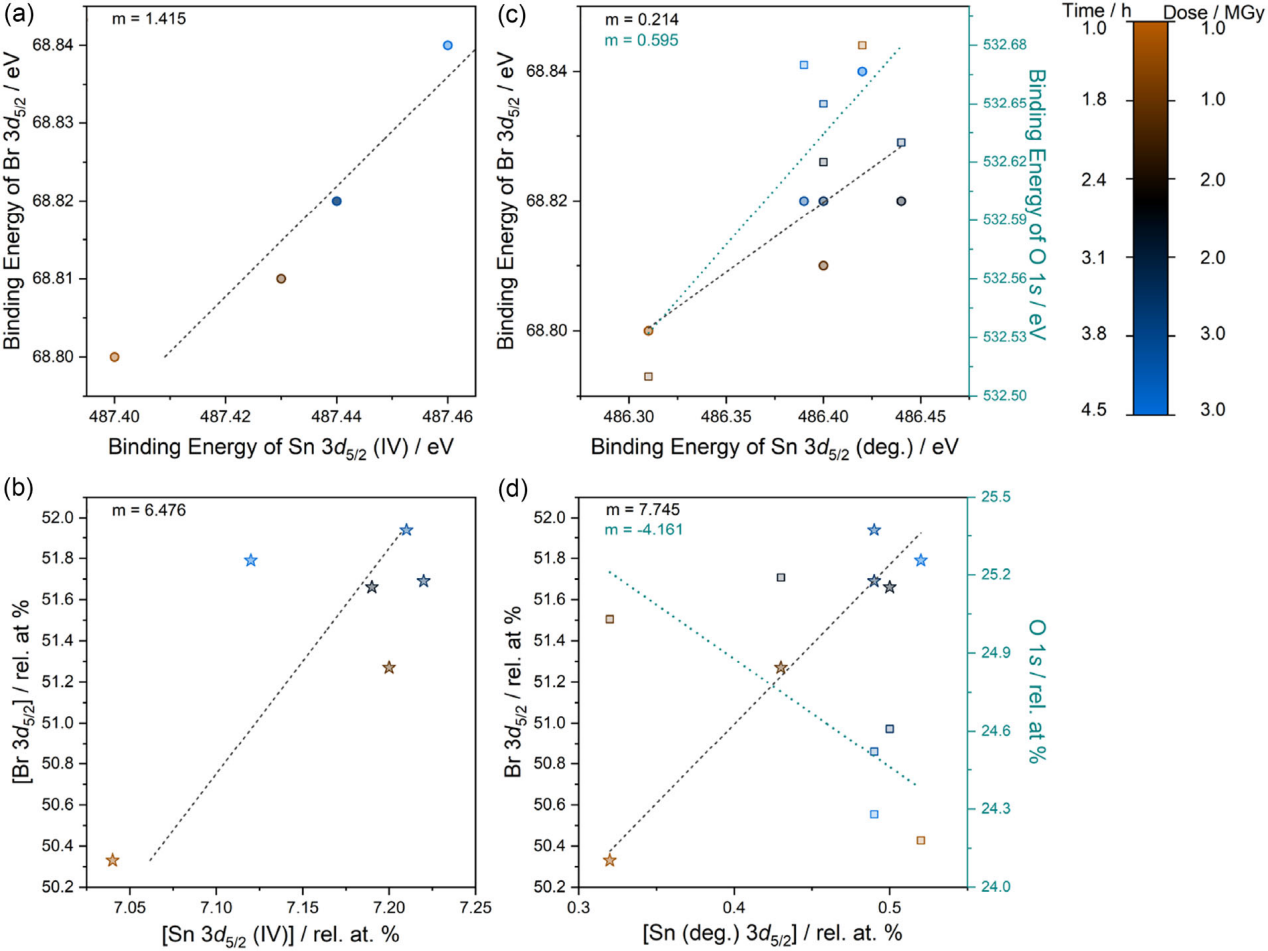
Correlation graphs a,c) BE positions and b,d) phase fractions from the XPS core‐level dataset of (NH_4_)_2_SnBr_6_ radiation damage experiment. Correlations are depicted for degraded peaks in XPS that have been assigned as a,b) Sn 3*d*_5/2_ (IV) against Br 3*d*_5/2_ and c,d) Br 3*d*_5/2_, shown with circular/starred scatter points and black gradient line, and O 1*s*, shown with square scatter points and a teal gradient line against degraded Sn 3*d*_5/2_ (deg.). Correlations of their BE positions and phase fractions are shown as a function of X‐ray exposure and values of the gradient (*m*) for these correlations are provided in each subfigure.

BE correlations in **Figure** **8**a show that Sn 3*d*_5/2_ (IV) and Br 3*d*_5/2_ (with *m* = 1.415) are within a single phase, in this case, (NH_4_)_2_SnBr_6_. The correlation of Br 3*d*_5/2_ and N 1*s* (*m* = 1.528), in Table S4, Supporting Information, further supports this. The phase fraction between Br 3*d*_5/2_ and Sn 3*d*_5/2_ (IV) is 6.476 in Figure 8b (compared to an expected value of 6 from the chemical formula), and between N 1*s* and Br 3*d*_5/2_ is 0.211 (compared to an expected value of 0.333 from the chemical formula), all confirming the double perovskite phase. The rel. at% values in Figure S12b, Supporting Information show a decrease in Br and N in the system, commensurate with the formation of NH_3_ and Br_2_ gas as observed for FA_2_SnBr_6_, the former also noted for MAPbI_3_.^[^ ^46^ ^]^

Although no immediately visible changes occur to the core‐level spectra during X‐ray irradiation, on closer inspection, an additional line broadening of the Sn 3*d* and Br 3*d* spectra in Figure 7a,b is noted. For the Sn 3*d*_5/2_ line the total FWHM increase is 0.13 eV, while for Br 3*d* it is 0.04 eV. Careful peak fit analysis and the addition of a second feature reveal a Sn (deg.) species. However, the addition of a Br (deg.) doublet in Br 3*d* resulted in compromised models that could not provide both mathematically and physically sensible doublet peaks and was therefore abandoned. Correlation analysis between Sn 3*d*_5/2_ (deg.), Br 3*d*_5/2_ (see Figure S8, Supporting Information for representative peak fits of both), and O 1*s* (see Figure S9, Supporting Information for the core level and its representative peak fit) were carried out. Br and O are able to form chemically stable phases with Sn, either as SnBr_*x*_ or SnO_*x*_.^[^ ^11^ ^]^ BE correlations of Sn with both Br and O are positive, with *m* = 0.214 (gray line in Figure 8c) and *m* = 0.595 (teal line in the same figure), respectively. However, neither are unitary relations. When BE correlations are linear but not unitary, this indicates that peak fitting protocols are unoptimized or the observed correlation is purely coincidental and not a reflection of a chemical relation, see Bhatt et al. for further details.^[^ ^19^ ^]^ Correlation of elemental quantification can help which scenario described is the case. If phase fractions are positive, the phase likely exists, but the peak fitting is not optimal. If phase fractions are negative, the phase itself is chemically illogical and the BE correlation observed is coincidental. Figure 8d shows the elemental compositional relations for O and Br with Sn (deg.). Whilst the O:Sn (deg.) phase fraction is negative (*m* = −4.161), the Br:Sn (deg.) phase fraction is positive (*m* = 7.745). The positive fraction with Br shows that as Sn (deg.) increases due to increasing irradiation, Br follows. In contrast, O decreases and, as seen in FA_2_SnBr_6_, is likely an adventitious species. In fact, all species that belong to the ammonium double perovskite increase as the experiment progresses, while there is a decreasing quantity of adventitious C and O in Figure S12b, Supporting Information that shows the elemental composition changes with irradiation. Thus, it is then possible that SnBr_*x*_ (*x* = 2 or 4) species form on the surface and that the peak fitting of Br 3*d* is not optimal.

It is difficult to differentiate between Sn (II) and Sn (IV) to identify SnBr_*x*_ as the BE difference between the two tin states is small, especially for perovskites and related structures.^[^ ^30^, ^47^ ^]^ A potential alternative is to focus on the peak analysis of Br 3*d*, whose failure has been described above. The most likely scenario is that the different Br 3*d* environments cannot be differentiated due to their BEs being almost identical. For (NH_4_)_2_SnBr_6_, irradiation results in the formation of a SnBr_*x*_ (*x* = 2 or 4) species and the NH_3_ and Br_2_ gases also seen in FA_2_SnBr_6_.

Following the exploration of undoped double perovskites, correlation analysis of compounds across the solid solution series can be undertaken. A key question is whether correlation analysis is able to show a difference in radiation‐induced changes hand‐in‐hand with a change of crystal structure from monoclinic to cubic.

#### 2.3.3 ((NH_4_)_(1−*x*)_FA_*x*_)_2_SnBr_6_, *x* = 0.03, 0.09

Within the ((NH_4_)_(1−*x*)_FA_*x*_)_2_SnBr_6_ series, the lowest and the highest amount of FA in the cubic ammonium structure were analytically determined from elemental analysis to be *x* = 0.03 and 0.09, respectively. Radiation‐induced damage for these compounds can be understood using the degradation pathways for the end members with *x* = 0, 1. Core‐level spectra during irradiation of ((NH_4_)_0.97_FA_0.03_)_2_SnBr_6_ in Figure S13, Supporting Information largely follow the evolution of spectra seen in (NH_4_)_2_SnBr_6_ above. Spectral features of Sn 3*d*, N 1*s*, and C 1*s* are largely unchanged over the course of the XPS experiment. The relative intensity of Sn 3*d*(deg.) to Sn 3*d*(IV) is much higher in the *x* = 0.03 compound than (NH_4_)_2_SnBr_6_, indicating differences in radiation stability even with compounds with the same crystal structure. The main difference for *x* = 0.03 is seen in Br 3*d*, where the peak width increases after exposure to 1.0 MGy of radiation by 0.12 eV.

Peak fitting for spectra collected after a maximum dose of 3.0 MGy is shown in Figure S10. The raw data from XPS are shown as blue scatter points, with the differently shaded peaks showing different spectral features in a given core level. The dashed gray line shows the Shirley background applied for peak fitting, and the orange line represents the overall envelope from the peak fitting procedures. The spectra show the formation of a Sn 3*d* (deg.) in Figure S10(a) and Br 3*d* (deg.) in Figure S10(b). Br (deg.) is seen at higher BE for both *x* = 0.03 and *x* = 0.09 (whose peak fits are in Figure S11, Supporting Information). The relative position of Br 3*d* (deg.) for doped compounds is different to the feature seen for FA_2_SnBr_6_, where it appeared at lower BE. It is also different to (NH_4_)_2_SnBr_6_, where no obvious secondary Br environment could be reasonably modeled. The formation of a Br (deg.) species is visually only observed in spectra where the formamidinium ion is present. The Br (deg.) phase also appears to be different between the undoped and doped formamidinium‐containing compounds.

Like before, correlation analysis of BE confirms that Sn (IV), Br, and both N 1*s* environments, namely, (N–H) and (N=C)/(N–C), belong to the double perovskite system, summarized in Table S4, Supporting Information. From the (deg.) species, Br 3*d*_5/2_ (deg.) and Sn 3*d*_5/2_ (deg.) have a positive BE correlation, *m* = 1.339. For FA_2_SnBr_6_, the Sn (deg.) with Br (deg.) environments were assigned to SnBr_2_. While in contrast, for (NH_4_)_2_SnBr_6_, they were assigned to SnBr_*x*_ (*x* = 2 or 4). For the doped sample *x* = 0.03, the phase fraction for Sn (deg.):Br (deg.) is 5.521. This differs from the formation of SnBr_2_ and SnBr_*x*_ species in the undoped samples and indicates the formation of SnBr_4_ for these compounds.

**Figure 9:**
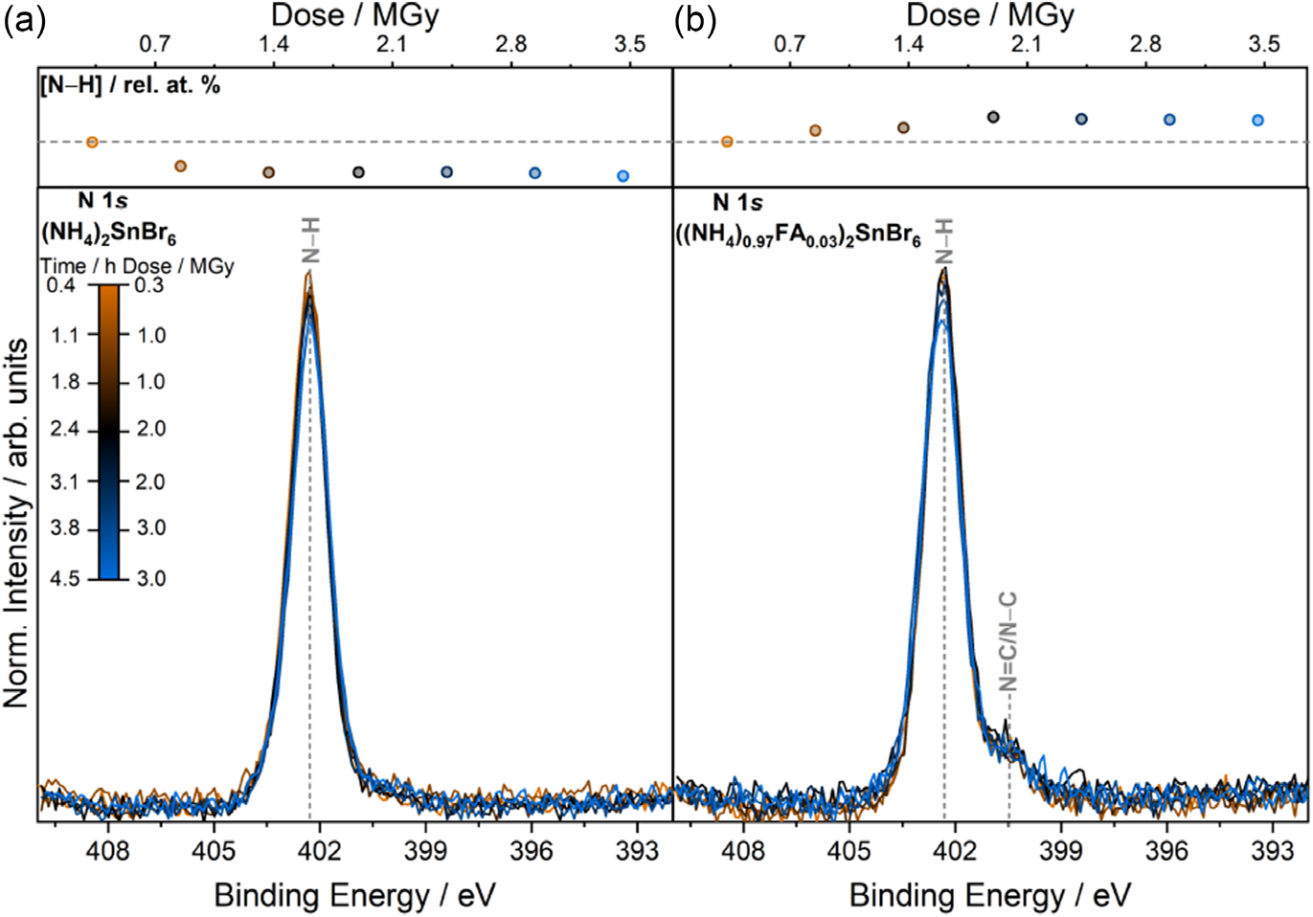
N 1*s* spectra of a) (NH_4_)_2_SnBr_6_ and b) ((NH_4_)_0.97_FA_0.03_)_2_SnBr_6_ as a function of X‐ray exposure. Data are normalized to the peak area of the Sn 3*d*_5/2_ spectra after exposure to 0.3 MGy of X‐ray radiation. For ease of visual comparison, spectra were aligned to the BE positions of the N 1*s* main spectral contribution at 0.3 MGy. The top panels show the quantification of the N–H environment, [N–H], (in rel. at%) with the gray dashed line representing the quantification at 0.3 MGy for reference.

Within the doped system, N 1*s* related correlations are more complex to disentangle. From the earlier correlation analyses in this work, it is understood that the degradation of FA results in the formation of an N (deg.) species that coincides with the BE of the N–H environment. To explore if the same degradation occurs for FA in the doped structures, **Figure** **9** compares the differences in the N–H environment in N 1*s* between *x* = 0 and *x* = 0.03 for ((NH_4_)_(1−*x*)_FA_*x*_)_2_SnBr_6_. In the figure, N 1*s* has been normalized to the area of the Sn 3*d*_5/2_ after exposure to 0.3 MGy dose. The top panels show the evolution of the N–H environment [N–H], in rel. at% over the course of the experiments.

In Figure 9a, the N–H environment is the only spectral feature in the N 1*s* core level. The formation of NH_3_(g) has been discussed above, and as a result of this, the N–H environment relative to Sn 3*d*_5/2_ diminishes. This is seen by the decrease in [N–H] as the scatter points fall below the gray dashed line in the plot in the top panel. In contrast, Figure 9b shows that [N–H] increases over the RADDAM experiment for ((NH_4_)_0.97_FA_0.03_)_2_SnBr_6_. Considering both compounds ((NH_4_)_0.97_FA_0.03_)_2_SnBr_6_ and (NH_4_)_2_SnBr_6_ contain the same NH_4_ environment, the expected trend for N–H should be a fall in [N–H] as NH_3_(g) forms. The difference for the doped compound can now be understood by considering the FA^+^ cation. For FA_2_SnBr_6_, the FA cation degrades to formamidine seen as a N (deg.) species in the XP spectra. The N (deg.) feature is known to coincide on the BE scale with the N–H environment for ammonium‐containing compounds. This N (deg.) feature from the FA cation, while difficult to visually separate from N–H contributions at the same BE position by peak fitting and thus correlation analysis, is now seen by the increasing [N–H] in comparison to Sn 3*d* during RADDAM. This shows that FA degradation is the same across all double perovskite structures, forming formamidine on the sample surface. The difference between the pure ammonium and the doped double perovskites is the formation of the additional N (deg.) species from the FA cation in the doped structure. Scrutiny of total elemental compositions in Figure S12c,d, Supporting Information also shows formation of NH_3_(g) and Br_2_(g) seen previously.

For the doped compounds in the solid solution series, the degradation of the Sn species to SnBr_4_ is different to FA_2_SnBr_6_ and possibly to (NH_4_)_2_SnBr_6_, while the degradation of the FA^+^ cation to formamidine is the same as in FA_2_SnBr_6_. The formation of NH_3_(g) and Br_2_(g) is consistent with all irradiation experiments conducted.

## 3 Conclusion

This work presents the synthesis and characterization of ((NH_4_)_(1−*x*)_FA_*x*_)_2_SnBr_6_ (*x* = 0, 0.03, 0.04, 0.06, 0.09, 1) as powders and crystals (for *x* = 1). For *x* = 1, formation of a new double perovskite is reported for the first time, and SCXRD revealed crystallization of the compound in the monoclinic space group P2_1_/*n*. This differs from the more common $\text{Fm}\bar{3}m$ cubic structure found for vacancy‐ordered double perovskites due to its large A‐site cation. Differences in the octahedral alignment as a result of the large A‐site cation size were discussed. Additionally, it was found that doped double perovskites adopted the cubic form and that a maximal dopant level of 9% FA into the (NH_4_)_2_SnBr_6_ structure type was achieved. Radiation damage using XPS was conducted, and effects were investigated for ((NH_4_)_(1−*x*)_FA_*x*_)_2_SnBr_6_ (*x* = 0, 0.03, 0.09, 1). Correlation analysis was used to assess the robustness of the peak fitting of various core levels to qualitatively understand degradation mechanisms across the solid solution series. The initial confirmation of the double perovskite from correlation analysis aids in understanding which spectral features arise due to radiation damage, and compare to degradation of other halide perovskites studied in literature. For FA_2_SnBr_6_, irradiation resulted in the formation of SnBr_2_, formamidine, NH_3_(g), and Br_2_(g). However, in (NH_4_)_2_SnBr_6_, a B‐site SnBr_*x*_ (*x* = 2 or 4) surface species forms, with the A‐ and X‐sites degrading to NH_3_(g) and Br_2_(g). Finally, for the doped compounds, Br_2_(g) formation was noted, and the Sn species was identified as SnBr_4_ indicating differences in Sn degradation for compounds with the same crystal structure. The FA cation degraded to formamidine and NH_3_(g) like in FA_2_SnBr_6_, and analysis of elemental concentration of the N–H was able to disentangle degradation pathways. It is clear that the B‐site degradation is heavily influenced by the chemical nature of the compound, which is not seen for the A‐ and X‐site. This may be attributed to the crystal structure and the type of A‐site cation of the double perovskite. The inclusion of the ammonium ion in the double perovskite likely increases the stability, as X‐ray degradation of the A‐site forms gaseous species, rather than formamidine that stays as a surface species for FA‐based compounds. These could likely promulgate the degradation of the double perovskite. This shows that a combination of robust peak fitting procedures and correlation analysis enables systematic degradation studies for a range of compounds. Achieving a comprehensive overview of the degradation pathways for perovskite‐like compounds is important to supplement studies of the material as photoabsorbers in photovoltaic devices.

## 4 Experimental Section

FA_2_SnBr_6_ was synthesized by solution‐phase co‐precipitation in air. An acidic solution of FABr was produced by dissolving 6.22 mmol formamidinium acetate C_3_H_8_N_2_O_2_, (Sigma Aldrich, 99%) in 3 mL of HBr (Sigma Aldrich, 48%). In parallel, 3.11 mmol SnBr_4_ (Sigma Aldrich, 98%) was dissolved in 3 mL of ethanol (Sigma Aldrich, 98%). The SnBr_4_ solution was added to the FABr solution, precipitating the double perovskite under stirring. The product was filtered under a vacuum, washed with ethanol, and dried in air. Single crystals were produced by solvent evaporation, with yellow crystals being observed after one week.

For (NH_4_)_2_SnBr_6_, the procedure is identical, except for the dissolution of 9.46 mmol NH_4_Br (Alfa Aesar, 99%) in 2 mL distilled water. To produce FA‐doped (NH_4_)_2_SnBr_6_ ((NH_4_)_(1−*x*)_FA_*x*_)_2_SnBr_6_, $\frac{\text{FA}}{\left(\text{FA}+{\text{NH}}_{4}\right)}/\left(x\right)=0.03,0.04,0.06,0.09$, the FABr and NH_4_Br solutions were mixed in the required proportions before adding the SnBr_4_ solution as described above. All samples appeared yellow, typical for tin (IV) bromide double perovskites.

PXRD was measured using an STOE Stadi‐P diffractometer (with STOE Dectris Mythen 1 K detector) employing a monochromated Cu K_*α*_ X‐ray source (*λ* = 1.5418 Å) with Debye–Scherrer geometry in transmission mode. Pawley refinement was carried out using the TOPAS‐Academic version 7.1 software suite for determination of lattice parameters.^[^ ^48^, ^49^ ^]^ Rietveld refinement of FA_2_SnBr_6_ was performed within the GSAS II software suite.^[^ ^50^ ^]^

SCXRD was performed using an Agilent SuperNova diffractometer with an Atlas CCD detector. Full spheres of data were collected using 1° scan frames in *ω* with Cu K_*α*_ radiation at 280 K (active temperature control). Data were processed with the CrysAlisPro 1.171.42.60a software package, and the structure was solved with SHELXT^[^ ^51^ ^]^ and refined with SHELXL^[^ ^52^ ^]^ within the Olex2 software suite.^[^ ^53^ ^]^

UV–visible spectroscopy was conducted using a Shimadzu Scientific Instruments UV‐2700i spectrophotometer in the integrated sphere mode. The spectrophotometer has a double monochromator. Excitation sources from deuterium and tungsten–halogen lamps have a switch‐over wavelength of 323 nm. Spectra were collected from 185 to 900 nm, with a step size of 2 nm and a dwell time of 50 ms. Samples were referenced against BaSO_4_ and were prepared by embedding powders on discs of pressed BaSO_4_ powder.

Elemental analysis (C, N, H) of the ((NH_4_)_(1−*x*)_FA_*x*_)_2_SnBr_6_ series was carried out using flash combustion of powders on a Thermo Scientific Flash 2000, Organic Elemental Analyzer. The machine was calibrated using an acetanilide standard (CE instruments, ≤100%). Detailed treatment of elemental analysis data to calculate the concentration of FA is presented in the Supporting Information, Section S3.

XPS experiments were performed on a Thermo Scientific NEXSA G2 spectrometer at HarwellXPS, Didcot, UK. The instrument employs a monochromatized Al K_*α*_ photon excitation source (*hν* = 1486.6 eV), a hemispherical analyzer, and a two‐dimensional detector. Measurements were conducted with a 400 μm spot size, and a dual Ar^+^ ion and electron source flood gun with 0.12 mA current, at 4.7 × 10^−7^ mbar. The photon flux at these experimental parameters is calculated to be ≈3.8 × 10^10^ photons s^−1^ from source parameters obtained from the manufacturer. Charge compensation was employed by a dual Ar ion and electron source flood gun. Pass energies of 200 and 40 eV were used to collect the survey and core‐level spectra, respectively. An experimental resolution of 572 meV was determined from a scrapped polycrystalline Au foil reference using the 16/84% standard method of resolution determination.^[^ ^54^ ^]^ Intensities are normalized to the area of the Sn 3*d*_5/2_ peak in each spectrum after removing a constant linear background. Binding energies have been referenced to Br 3*d*_*5/2*_ at 68.7 eV, in the discussion on the solid solution series of ((NH_4_)_(1−*x*)_FA_*x*_)_2_SnBr_6_. All other spectra are left uncorrected for correlation analysis. Br 3*d*_5/2_ alignment is chosen over typical carbon corrections as adventitious C^0^ overlaps with C–H from FA in C 1*s*. This has been addressed for organic‐inorganic compounds elsewhere.^[^ ^28^ ^]^ X‐ray irradiation dose absorbed by the crystalline samples during XPS experiments was determined using the RADDOSE‐3D version 4 utility, developed by Garman et al.^[^ ^55^, ^56^ ^]^ X‐ray dose was estimated using the average dose whole crystal (AD‐WC) metric, previously applied by Fernando et al.^[^ ^16^ ^]^ for XPS experiments. Dose values have been rounded to the nearest whole number, as applied by Fernando et al. previously.^[^ ^16^, ^33^, ^57^ ^]^ Input parameters are detailed in Section S4, Supporting Information.

## Conflict of Interest

The authors declare no conflict of interest.

## Supporting information

Supplementary Material

## Data Availability

Data for this article, including all processed data of the main paper figures, are available at Zenodo in Origin format at [https://doi.org/10.5281/zenodo.16759005]. Deposition Number 2417239 (https://www.ccdc.cam.ac.uk/services/structures?id=doi:10.1002/cphc.uk202500370) (for FA_2_SnBr_6_), contains the supplementary crystallographic data for this paper. These data are provided free of charge by the joint Cambridge Crystallographic Data Centre and Fachinformationszentrum Karlsruhe Access Structures service (https://www.ccdc.cam.ac.uk/structures/).
