# Supplementary material for: Exploring the Radiation Damage of Vacancy‐Ordered Double Perovskites, ((NH4)(1−x)FA x )2SnBr6
Source: Chemphyschem. 2025 Nov 4;26(23):e202500370. doi: 10.1002/cphc.202500370 (PMC12677720; doi:10.1002/cphc.202500370)
Supplement: Supplementary file 1 — Supplementary Material [file CPHC-26-e202500370-s001.pdf]

Supplementary Information
Exploring the Radiation Damage of Vacancy-Ordered Double Perovskites, $((NH_4)_{(1-x)}FA_x)_2SnBr_6$[†]

Prajna Bhatt,[a,†,‡] Theo Stucky De Quay,[a,†] Yuhan Liu,[a,b] Tim Evans,[a] Orfhlaith McCullough,[c] Nathalie K. Fernando,[a] Curran Kalha,[a] Robert G. Palgrave,[a] and Anna Regoutz[a,d,*]

[a] *Department of Chemistry, University College London, 20 Gordon Street, London, WC1H 0AJ, UK.*

[b] *The Electrochemical Innovation Lab, Department of Chemical Engineering, University College London, WC1E 7JE, UK.*

[c] *School of Human Sciences, Science Centre, London Metropolitan University, London, N7 7DD, UK.*

[d] *Department of Chemistry, University of Oxford, Inorganic Chemistry Laboratory, South Parks Road, OX1 3QR, Oxford, UK.*

[†] Both authors contributed equally to the manuscript.

[‡] *Current address: Istituto Officina dei Materiali (IOM)-CNR, Laboratorio TASC, in Area Science Park, S.S.14, Km 163.5, Trieste I-34149, Italy.*

Corresponding author: [*] *anna.regoutz@chem.ox.ac.uk*

1 Rietveld refinement of FA_2SnBr_6

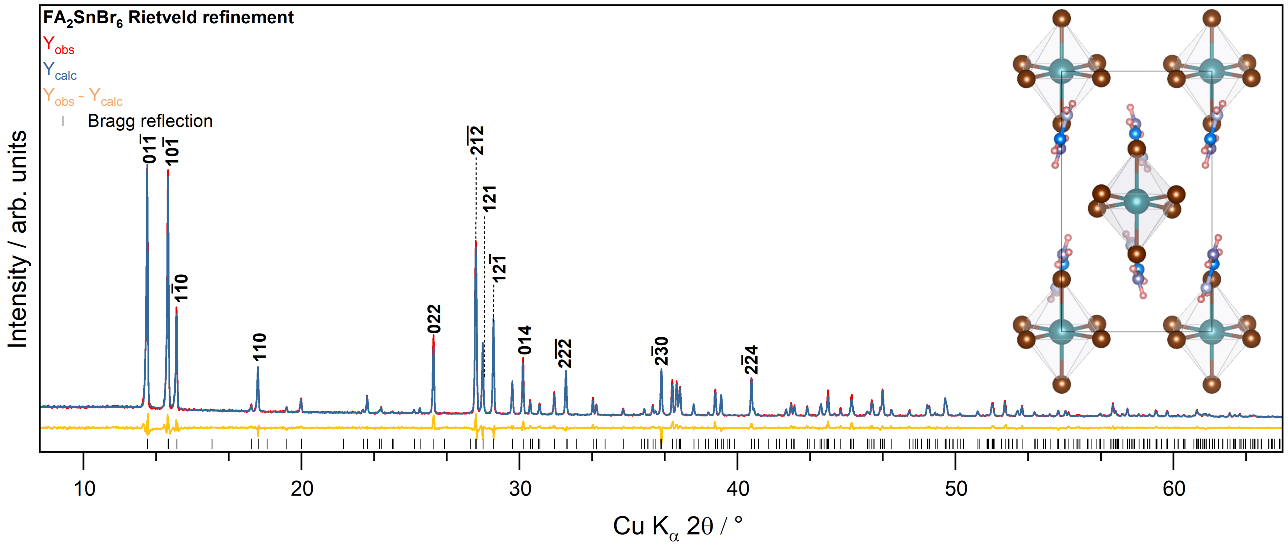

Figure S1: Rietveld refinement of PXRD data of FA_2SnBr_6. Lattice parameters were obtained and refined using SCXRD. Refinement values are R_w = 9.676% and GOF = 1.16. The inset shows the crystal structure of the compound along the c-axis, made using the VESTA version 3 software package.[1] Brown atoms represent Br, pink atoms represent H, blue atoms represent C, purple atoms represent N, and teal atoms represent Sn. The light grey octahedra represent $[SnBr_6]^{2-}$.

2 Exploration of solid solution series $((NH_4)_{(1-x)}FA_x)_2SnBr_6$ with PXRD

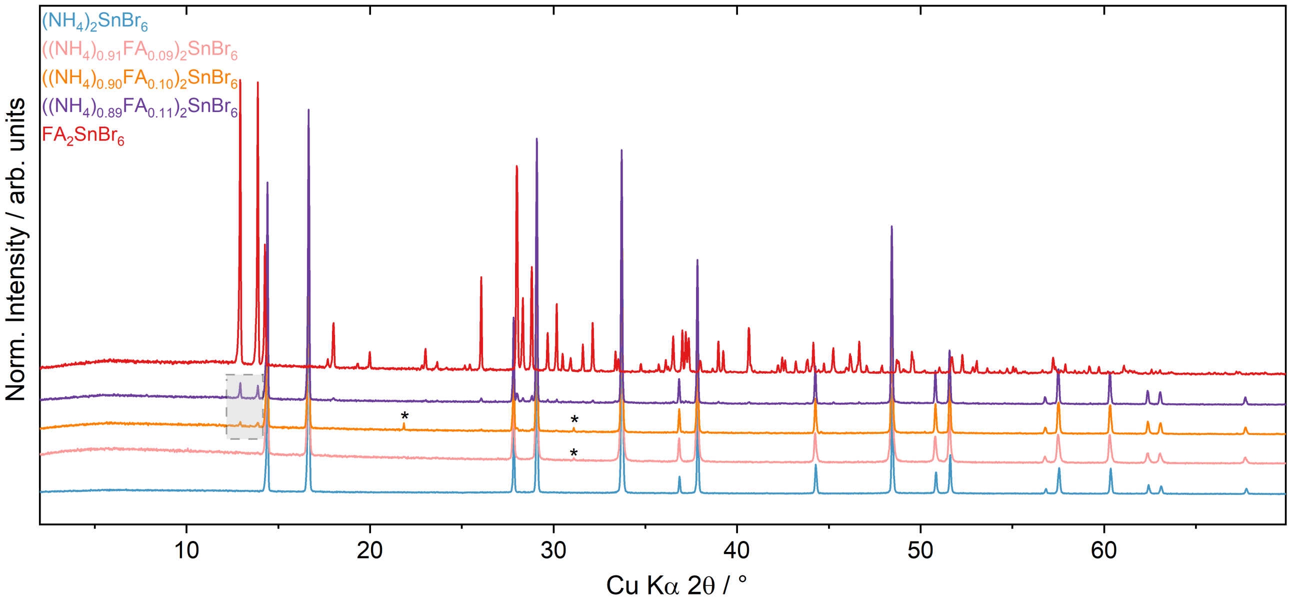

Figure S2: PXRD patterns of $((NH_4)_{(1-x)}FA_x)_2SnBr_6$ with x = 0, 0.09, 0.10, 0.11, and 1. The grey box highlights reflections that belong to the x = 1 pattern and indicate the formation of multiple perovskite phases with x ≥ 0.10. Asterisks mark reflections from NH_4Br.

3 Doping content x in $((NH_4)_{(1-x)}FA_x)_2SnBr_6$

In order to understand the bulk doping quantity of FA into the cubic $(NH)_4SnBr_6$ structure, weight % (wt. %) data from elemental analysis (EA) were treated as follows. For wt. % of carbon, the FA ion would be the sole contribution to the presence of carbon in the crystalline powder. The minimally detected wt. % of carbon in $(NH_4)_2SnBr_6$ (0.04 %) was subtracted from % carbon in the rest of the doped series and normalised to the expected wt. % of carbon in an FA ion (3.49 %). In the case of wt. % nitrogen, the nitrogen contribution from the ammonium ion (4.42 %) is subtracted from all data values in the doped series and normalised to the expected wt. % difference between the formamidinium and ammonium ion (3.72 %). Figure S3 plots the x values obtained from EA using nitrogen against x from EA using carbon and XPS using N 1*s*.

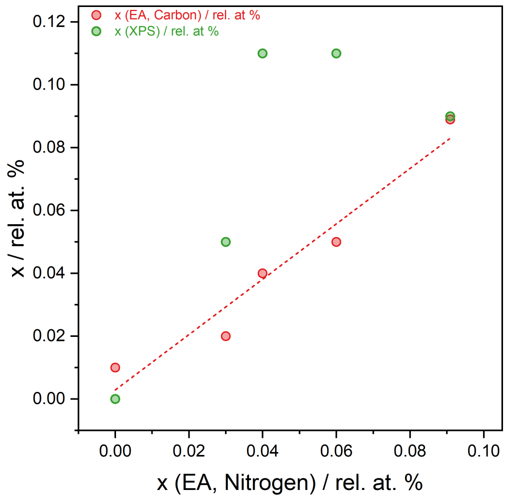

Figure S3: Concentration of FA x in $((NH_4)_{1-x}FA_x)_2SnBr_6$ calculated from elemental analysis (EA) of the nitrogen and carbon components and XPS quantification in rel. atomic %.

4 X-ray dose estimation using RADDOSE-3D

Table S1: The parameters included in the RADDOSE-3D input file for the small molecule feature used to estimate the dose during RADDAM experiments using a Thermo Scientific NEXSA G2 spectrometer.

RADDOSE Parameter	Thermo Scientific NEXSA G2
Crystal type	cuboid
Crystal Dimension / μm	$60 \times 60 \times 60$
PixelsPerMicron	1.6
Beam type	Gaussian
Photon flux / photons/s	3.80×10^{10}
FWHM / μm	326×579
Energy / keV	1.487
Collimation type	circular
Collimation dimensions / μm^2	400×710

The dose absorbed during XPS experiments was estimated using the average dose whole crystal (AD-WC) metric.

5 Doped series $((NH_4)_{(1-x)}FA_x)_2SnBr_6$ (x = 0, 0.03, 0.04, 0.06, 0.09

Figure S4 shows the bandgap extracted using the Kubelka-Munk and Tauc methods for the series. The trend in the bandgap for the solid solution series does not change and has been described in detail in the main paper.

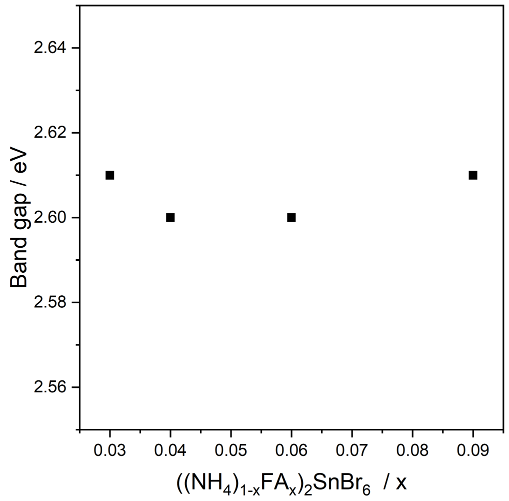

Figure S4: Band gap (E_g) of the $(((NH_4)_{1-x}FA_x)_2SnBr_6$ series measured using powders via diffuse reflectance spectroscopy between 185-900 nm.

Synthesis of the doped series was done across a wide range of nominal doping ratios, x = 0.25 to 0.9. The bulk elemental analysis described in Section 3.2 in the main text uses the analytical values of doping, which are much lower than the nominal values of the compound. Table S2 shows the difference between the nominal and analytical x values determined using elemental analysis.

Table S2: Nominal and analytical FA amounts (x) in the doped series, $(NH_4)_{1-x}FA_x)_2SnBr_6$. Analytical amounts have been taken from combustion elemental analysis, which is described in the main paper and in the next Section.

Nominal x	Analytic x
0.5	0.03
0.6	0.04
0.65	0.06
0.80	0.09

6 Survey, core level, and valence state spectra of formamidinium-doped ammonium tin bromide, $((NH_4)_{(1-x)}FA_x)_2SnBr_6$

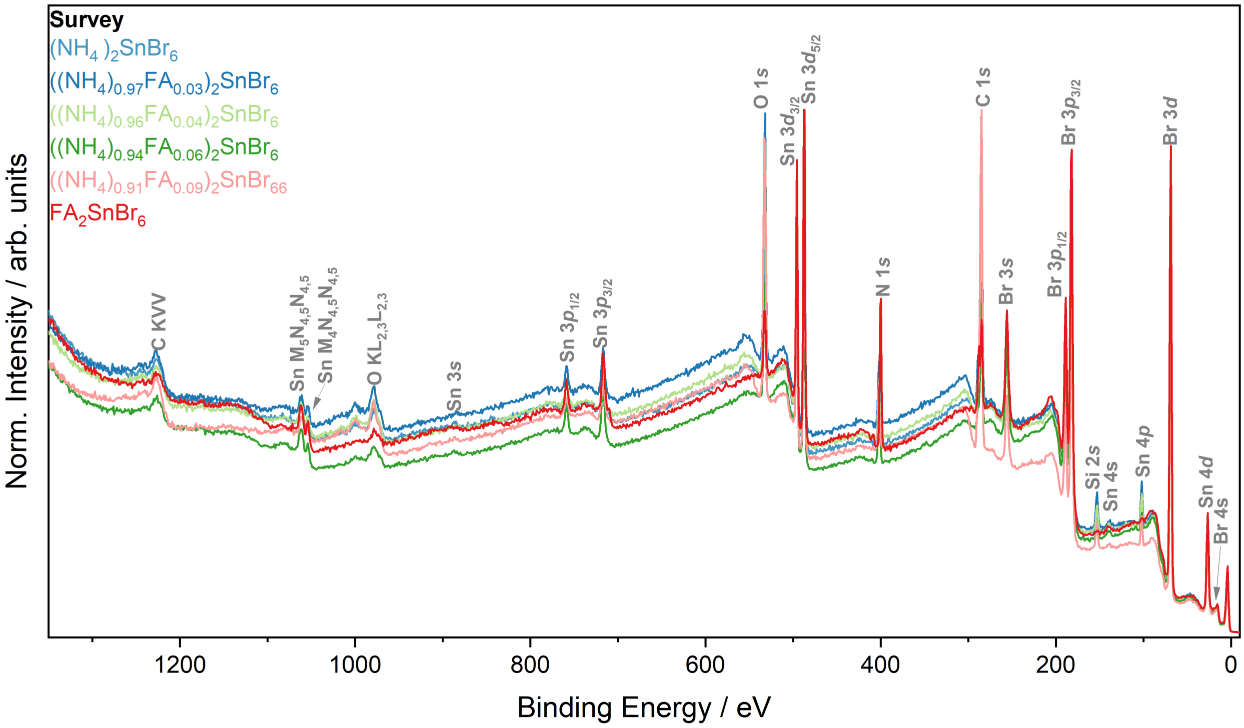

Figure S5: X-ray photoelectron survey spectra of the $((NH_4)_{1-x}FA_x)_2SnBr_6$ series. Data is normalised to the maximum peak height of each spectrum.

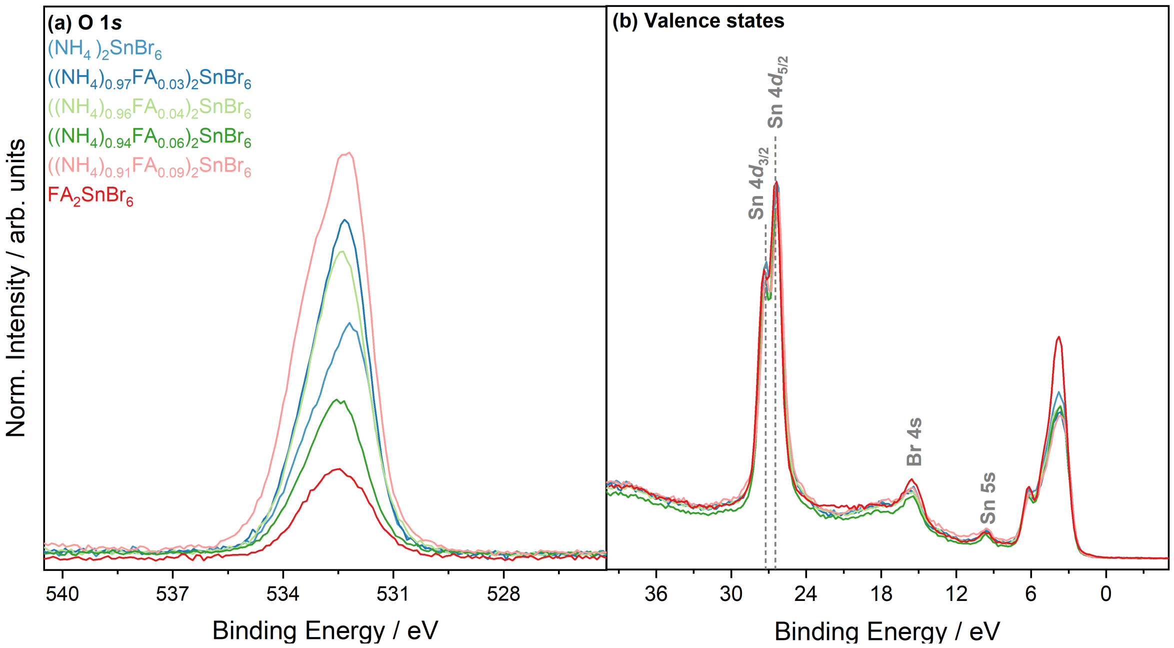

Figure S6: X-ray photoelectron spectra of the $((NH_4)_{1-x}FA_x)_2SnBr_6$ series, including the (a) O 1*s* and (b) valence states. Data is normalised to peak area of the respective Sn $3d_{5/2}$ spectra.

7 Peak fitting procedures and parameters

Peak fitting procedures for all compounds studied using XPS follow the same process, beginning with Sn 3*d*. All procedures were carried out using the Thermofischer Scientific Avatange software suite. Peaks were fit using pseudo-Voigt functions as sum of Gaussian (G) and Lorentizan (L) contributions. The G:L ratio was 70:30 for all peaks. All parameters except for peak position, area, height, and FWHM are left restricted on Avantage, to the software's default peak fitting restrictions. These include parameters that describe asymmetry in peak shapes namely tail mix, tail height and tail exponent. Restrictions to FWHM are applied after scrutiny with chemical understanding, briefly explained for Sn 3*d* below.

Consider FA_2SnBr_6 for which the peak fitting in Figure S7 is shown for spectra collected after 4 MGy X-ray dose exposure. In Figure S7(a) the peak arising from Sn (IV) is sharper than the Sn (deg.) peak, and has a lower FWHM. Chemically, this may be reasonable to assume as the Sn (deg.) peak can be from more than one species, resulting in the broadening of the peak. Care must be taken not to over-fit, so Sn (deg.) was initially fit as a singular peak. The FWHM was restricted for both Sn (IV) and Sn (deg.) peaks and is summarised in Table S3. A similar logic was applied to N 1*s*, in Figure S7(b) where the changes in spectra are visually obvious. For Br 3*d*, in Figure S7(c) the doublets were fit with a spin-orbit splitting (SOS) of 1.04 eV that falls within previously reported SOS values in the literature.[2,3] The increase in FWHM after 1 MGy of X-ray dose in the spectra suggests the formation of a secondary doublet, which fit with a second doublet environment at lower binding energies. C 1*s* is quite complicated, and so is avoided in discussions - as explained in the main paper. For sake of completeness, C 1*s* peaks were fit using methods applied for C 1*s* in correlation analysis for polymers elsewhere.[4] The FWHM were constrained for all peaks to the same value (1.20 eV). Over-fitting was avoided due to the presence of adventitious carbon that overlaps with the FA environments.

In the pure and doped $(NH_4)_2SnBr_6$ compounds (Figure S8,S11), the core levels are unchanging, relative to the damage seen in the formamidinium analogue. Only Sn 3*d* is fitted with a (deg.) feature. For the doped compounds, the same FWHM of the pure ammonium compound was used, as the compounds crystallised in the $(NH_4)_2SnBr_6$ structure type and mostly contain the NH_4 A-site. Applying fit parameters of FA_2SnBr_6 resulted in ill-fitting peaks, where the chemistry is inconsistent with the findings of the bulk structure from diffraction and elemental analysis.

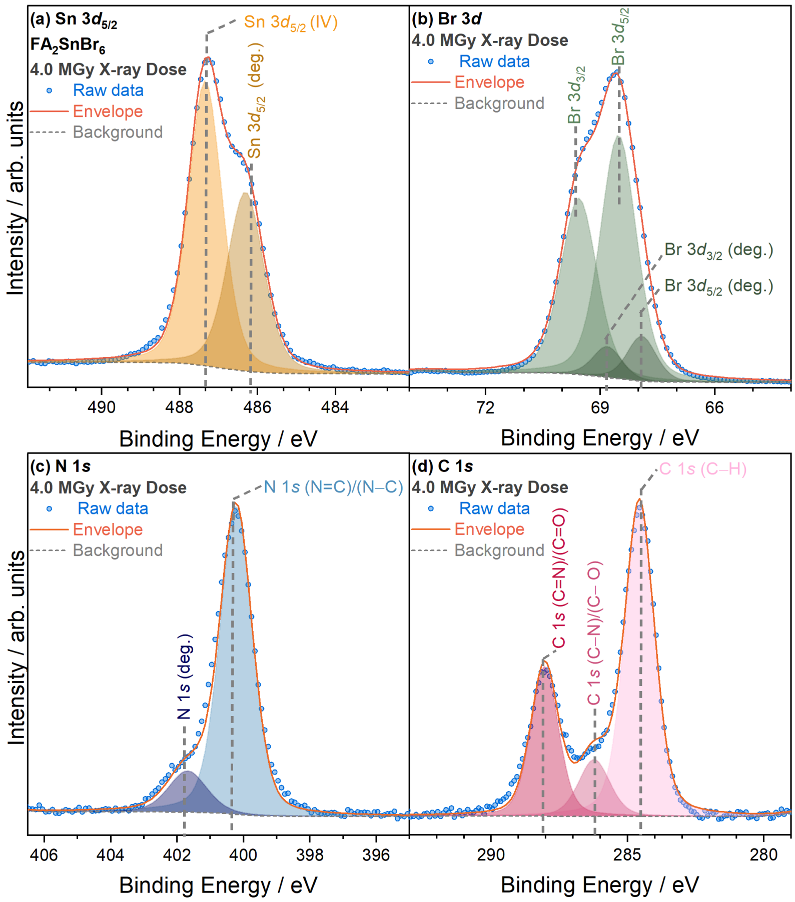

Figure S7: Peak fit of core level X-ray photoelectron spectra of FA_2SnBr_6 after exposure to 4 MGy of X-ray doe, including the (a) Sn 3$d_{5/2}$, (b) Br 3d, (c) N 1s, and (d) C 1s spectra. The raw data from XPS are shown as blue scatter points, with the differently coloured peaks showing different spectral features in a given core level. The dashed grey line shows the Shirley background applied for peak fitting, and the orange line represents the overall envelope from the peak fitting procedures. All procedures were done using the Avantage Software suite.

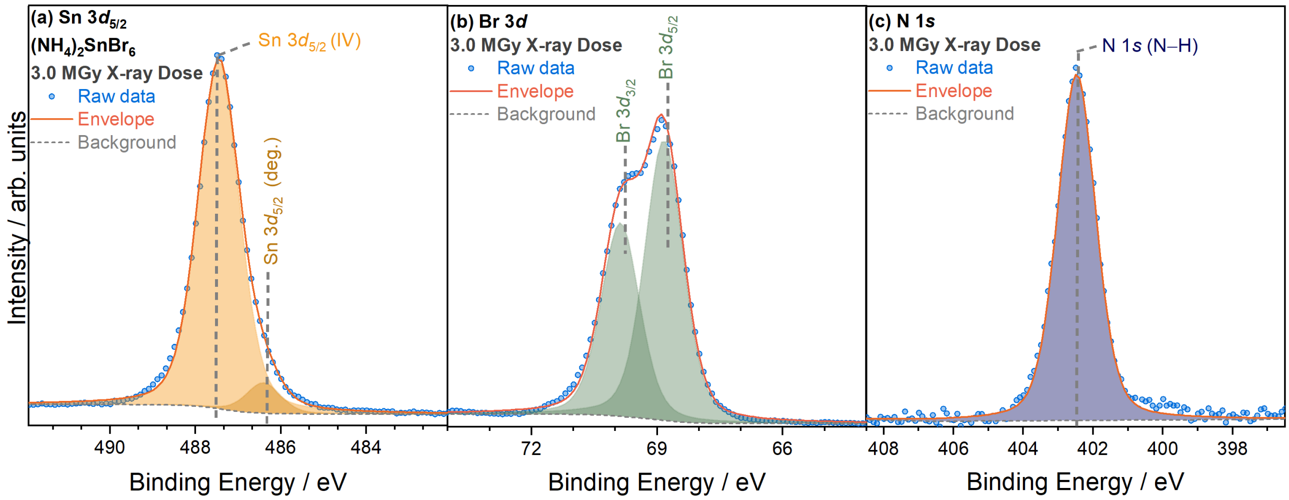

Figure S8: Peak fit of core level X-ray photoelectron spectra of $(NH_4)_2SnBr_6$ after exposure to 3 MGy of X-ray dose, including the (a) Sn 3$d_{5/2}$, (b) Br 3d, and (c) N 1s spectra. The raw data from XPS are shown as blue scatter points, with the differently coloured peaks showing different spectral features in a given core level. The dashed grey line shows the Shirley background applied for peak fitting, and the orange line represents the overall envelope from the peak fitting procedures. All procedures were done using the Avantage Software suite.

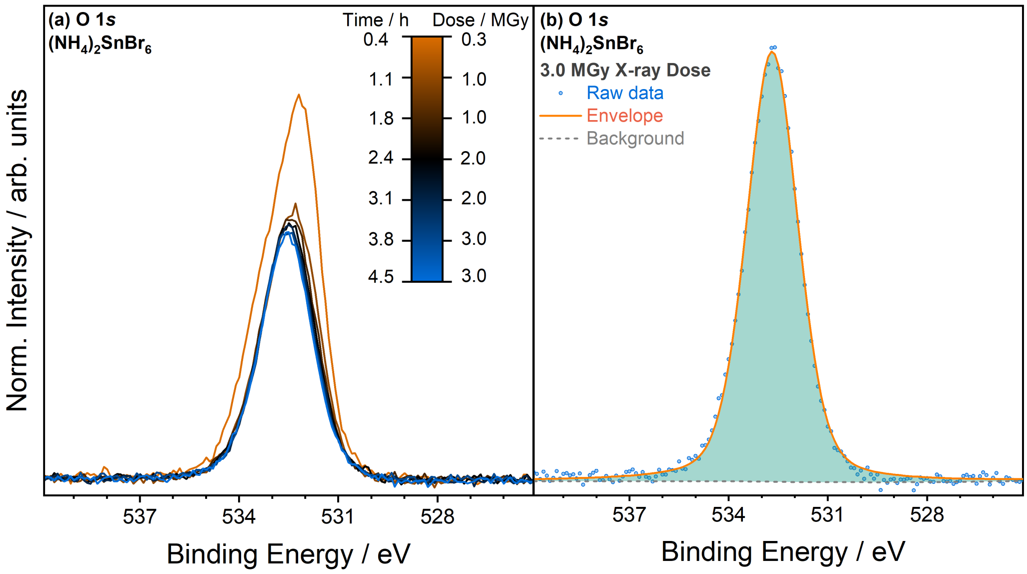

Figure S9: (a) O 1s core level X-ray photoelectron spectra of $(NH_4)_2SnBr_6$ as a function of X-ray exposure. Data is normalised to the peak area of Sn 3$d_{5/2}$ from the spectrum at 0.3 MGy of X-ray dose. (b) Peak fit of O 1s core level X-ray photoelectron spectra of $(NH_4)_2SnBr_6$ after exposure to 3 MGy of X-ray dose.

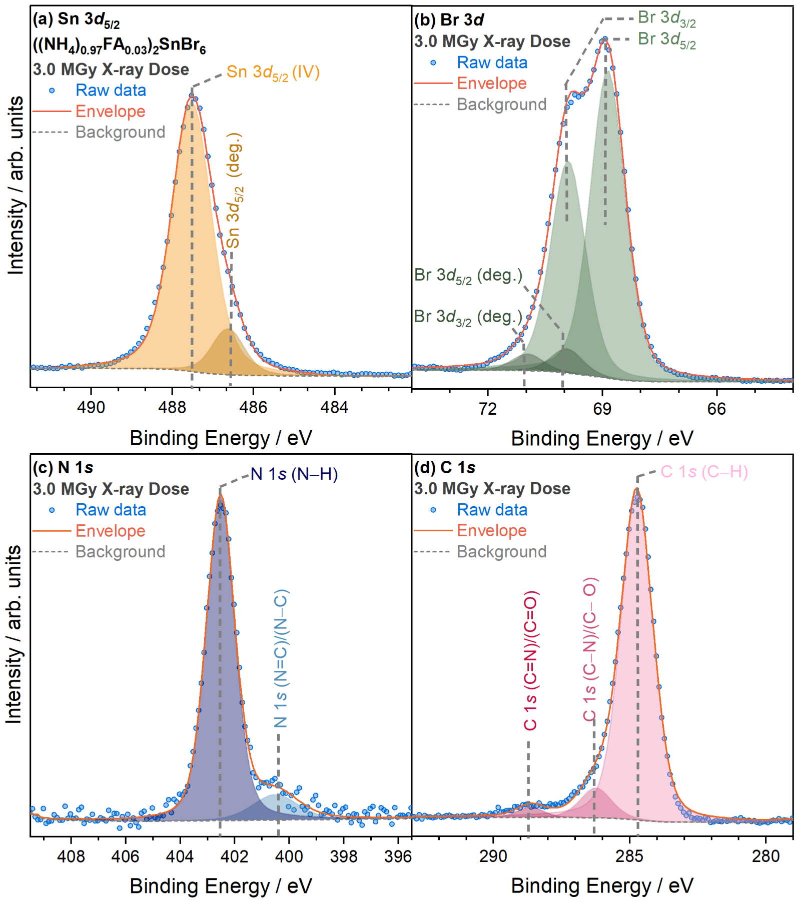

Figure S10: Peak fit analysis of core level X-ray photoelectron spectra of $((NH_4)_{0.97}FA_{0.03})_2SnBr_6$ after a maximum X-ray dose of 3.0 MGy, including the (a) Sn 3$d_{5/2}$, (b) Br 3d, (c) N 1s, and (d) C 1s spectra. The raw data from XPS are shown as blue scatter points, with the differently coloured peaks showing different spectral features in a given core level. The dashed grey line shows the Shirley background applied for peak fitting, and the orange line represents the overall envelope from the peak fitting procedures. All procedures were done using the Avantage Software suite.

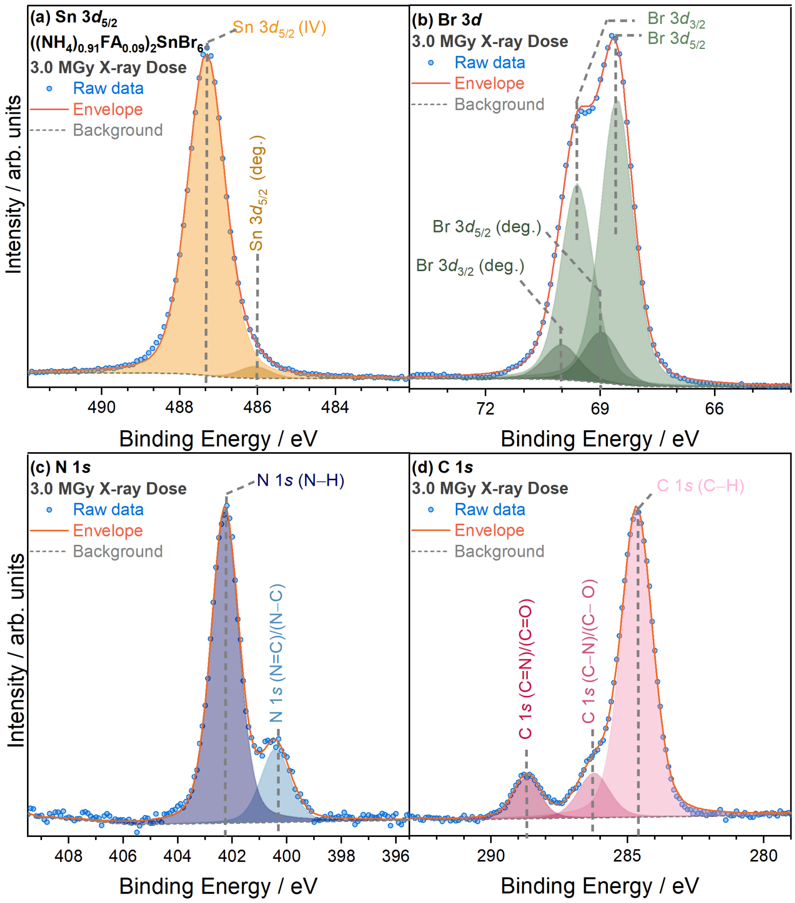

Figure S11: Peak fit of core level X-ray photoelectron spectra of 9% FA doped $(NH_4)_2SnBr_6$ after exposure to 3 MGy of X-ray dose, including the (a) Sn 3$d_{5/2}$, (b) Br 3d, (c) N 1s, and (d) C 1s spectra. The raw data from XPS are shown as blue scatter points, with the differently coloured peaks showing different spectral features in a given core level. The dashed grey line shows the Shirley background applied for peak fitting, and the orange line represents the overall envelope from the peak fitting procedures. All procedures were done using the Avantage Software suite.

Table S3: Data derived by fitting the Sn $3d_{5/2}$, Br $3d$, N $1s$ and C $1s$ core line photoelectron spectra to various pseudo-Voigt functions, across the $((NH_4)_{1-x}FA_x)_2SnBr_6$ series. Values of the full width at half maximum height (FWHM / eV), and spin orbit splitting (Δ_{SOS} / eV) are provided.

Sample	Core level assignment	FWHM / eV	Δ_{SOS} / eV
FA_2SnBr_6	Sn $3d_{5/2}$ (IV)	1.03	–
	Sn $3d_{5/2}$ (deg.)	1.11	–
	Br $3d$	1.12	1.04
	Br $3d$ (deg.)	0.84	1.04
	N $1s$ (N–C)	1.16	–
	N $1s$ (deg.)	1.36	–
	C $1s$	1.20	–
$(NH_4)_2SnBr_6$	Sn $3d_{5/2}$ (IV)	1.15	–
	Sn $3d_{5/2}$ (deg.)	0.95	–
	Br $3d$	1.09	1.04
	N $1s$ (N–H)	1.24	–
	O $1s$	1.84	–
$((NH_4)_{1-x}FA_x)_2SnBr_6$ (x = 0.03, 0.09)	Sn $3d_{5/2}$ (IV)	1.15	–
	Sn $3d_{5/2}$ (deg.)	0.95	–
	Br $3d$	1.09	1.04
	Br $3d$ (deg.)	0.98	1.04
	N $1s$ (N–C)	1.57	–
	N $1s$ (N–H)	1.24	–
	C $1s$	1.20	–

8 Radiation damage

In Figure S12 (d), only two data points are shown for the quantification for each core level, and only two levels of spectra were collected at 0.3 and 3 MGy X-ray dose, respectively. The higher FA doping experiment is only used in discussions along with the 3 % system to verify the degradation route in the doped series.

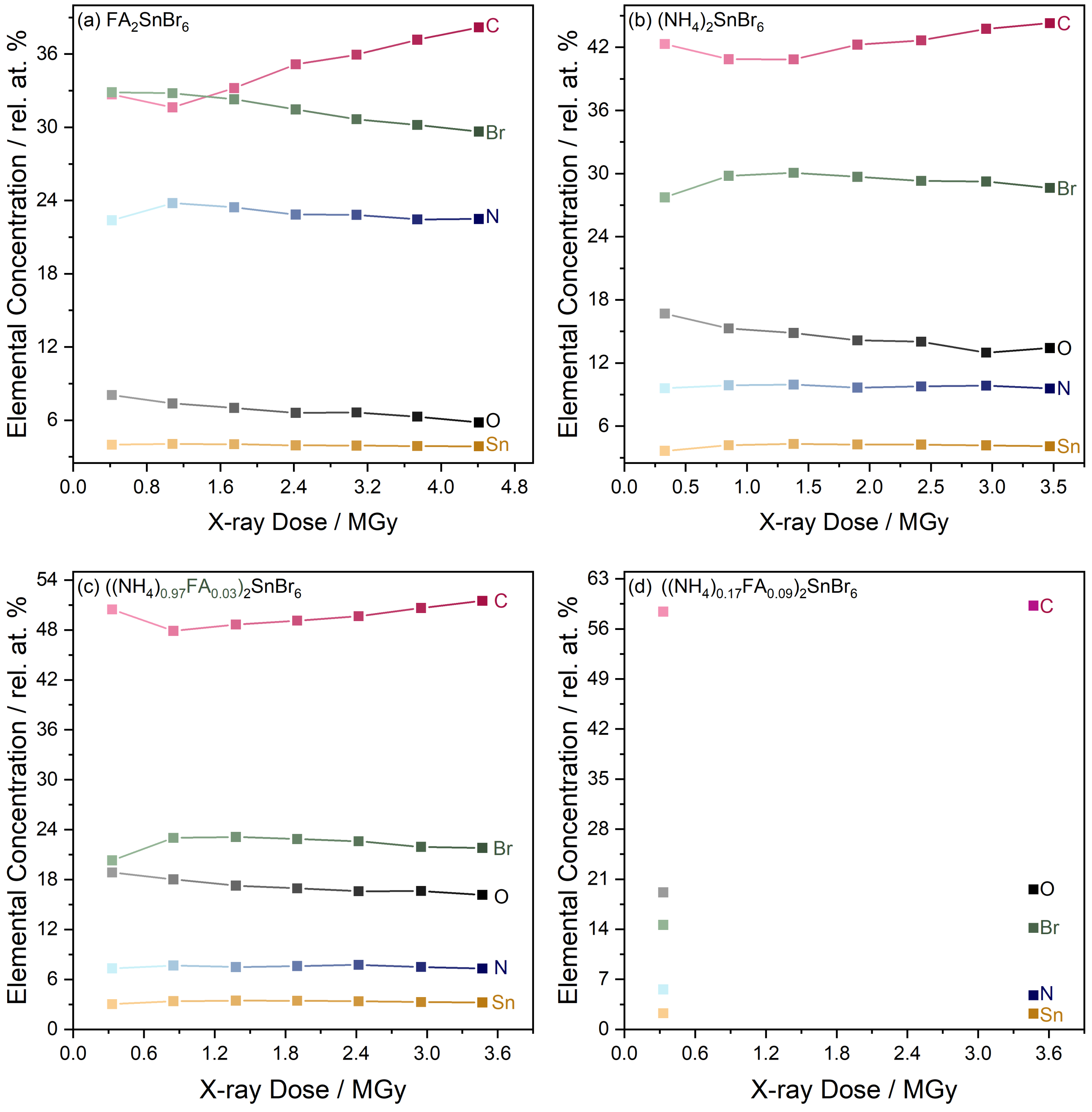

Figure S12: Elemental concentrations, in relative atomic percentages of $((NH_4)_{1-x}FA_x)_2SnBr_6$ determined from peak fit analysis of the main core state spectra (Br 3d, Sn 3d, N 1s, C 1s, and O 1s), for x = 0, 0.03, 0.09, and 1 (a-d) as a function of X-ray dose during XPS measurements.

Table S4: Correlation analysis derived from uncorrected binding energy positions and rel. at.% by peak fitting the Sn $3d_{5/2}$, Br $3d$, N $1s$, and C $1s$ core line photoemission spectra to various pseudo-Voigt functions, across the $((NH_4)_{1-x}FA_x)_2SnBr_6$ series. Gradient values are provided for relations not plotted in the main paper.

Sample	Correlation x vs. y	BE gradient / m	Rel. at.% gradient / m
FA_2SnBr_6	Sn $3d_{5/2}$ (IV) vs. N $1s$ (N=C)/(N−C)	0.983	2.804
	Br $3d_{5/2}$ vs. N $1s$ (N=C)/(N−C)	0.913	0.639
$(NH_4)_2SnBr_6$	Sn $3d_{5/2}$ (IV) vs. N $1s$	0.948	0.255
	Br $3d_{5/2}$ vs. N $1s$	1.528	0.211
	Sn $3d_{5/2}$ (deg.) vs. O $1s$	0.718	-4.160
$((NH_4)_{1-x}FA_x)_2SnBr_6$ (x = 0.03)	Sn $3d_{5/2}$ (IV) vs. N $1s$ (N-H)	1.03	2.345
	Sn $3d_{5/2}$ (IV) vs. Br $3d_{5/2}$	1.11	5.631
	Sn $3d_{5/2}$ (IV) vs. N $1s$ (N=C)/(N−C))	0.756	0.447
	Br $3d_{5/2}$ vs. N $1s$ (N=C)/(N−C)	0.778	0.132

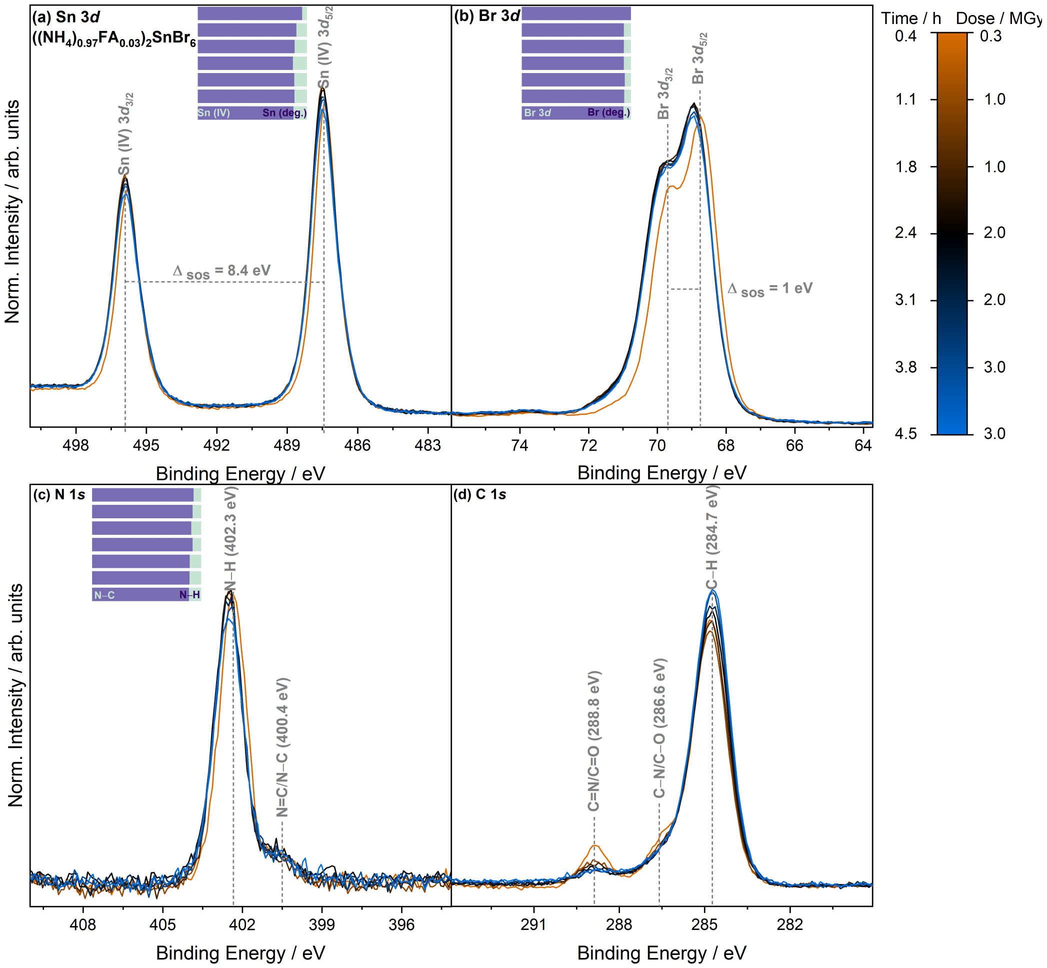

Figure S13: Core level X-ray photoelectron spectra of $((NH_4)_{0.97}FA_{0.03})_2SnBr_6$ as a function of X-ray dose, including (a) Sn $3d$, (b) Br $3d$, (c) N $1s$, and (d) C $1s$. The colour bar legend shows the cumulative measurement time and the calculated X-ray dose from the average dose of whole crystal (AD-WC) using RADDOSE-3D. The time given represents the end of each measurement period, with each data acquisition taking approximately 0.42 h. The grey dotted lines and BE values shown correspond to the positions of the visually discernible spectral features. The (deg.) label is used to describe spectral features that appear in core level spectra as a result of irradiation. Data are normalised to the peak area of Sn $3d_{5/2}$ from the spectrum after exposure to 0.3 MGy of X-ray dose after subtraction of a linear background. An indication of selected chemical species ratios as determined from peak fit analysis is included as a bar chart on the top left section of each subfigure.

References

[1] K. Momma and F. Izumi, *Journal of Applied Crystallography*, 2011, **44**, 1272–1276.

[2] K. Sun, O. J. Silveira, Y. Ma, Y. Hasegawa, M. Matsumoto, S. Kera, O. Krejčí, A. S. Foster and S. Kawai, *Nature Chemistry*, 2023, **15**, 136–142.

[3] L. Smykalla, P. Shukrynau, M. Korb, H. Lang and M. Hietschold, *Nanoscale*, 2015, **7**, 4234–4241.

[4] P. Bhatt, M. Isaacs, Y. Liu and R. G. Palgrave, *Applied Surface Science*, 2024, **672**, 160808.
